# The clinical drug candidate anle138b binds in a cavity of lipidic α-synuclein fibrils

**DOI:** 10.1038/s41467-022-32797-w

**Published:** 2022-09-14

**Authors:** Leif Antonschmidt, Dirk Matthes, Rıza Dervişoğlu, Benedikt Frieg, Christian Dienemann, Andrei Leonov, Evgeny Nimerovsky, Vrinda Sant, Sergey Ryazanov, Armin Giese, Gunnar F. Schröder, Stefan Becker, Bert L. de Groot, Christian Griesinger, Loren B. Andreas

**Affiliations:** 1 NMR-based Structural Biology, Max Planck Institute for Multidisciplinary Sciences, Göttingen, Germany; 2 Department of Theoretical and Computational Biophysics, Max Planck Institute for Multidisciplinary Sciences, Göttingen, Germany; 3 Institute of Biological Information Processing (IBI-7: Structural Biochemistry), Forschungszentrum Jülich, Jülich, Germany; 4 Department of Molecular Biology, Max Planck Institute for Multidisciplinary Sciences, Göttingen, Germany; 5 MODAG GmbH, Mikroforum Ring 3, 55234 Wendelsheim, Germany; 6 Center for Neuropathology and Prion Research, Ludwig-Maximilians-University Munich, Munich, Germany; 7 Physics Department, Heinrich Heine University Düsseldorf, Düsseldorf, Germany; 8 Cluster of Excellence “Multiscale Bioimaging: From Molecular Machines to Networks of Excitable Cells” (MBExC), University of Göttingen, Göttingen, Germany; 9 Present Address: Max Planck Institute for Chemical Energy Conversion, Mülheim an der Ruhr, Germany

**Keywords:** Solid-state NMR, Molecular modelling, Molecular neuroscience, Protein aggregation

## Abstract

Aggregation of amyloidogenic proteins is a characteristic of multiple neurodegenerative diseases. Atomic resolution of small molecule binding to such pathological protein aggregates is of interest for the development of therapeutics and diagnostics. Here we investigate the interaction between α-synuclein fibrils and anle138b, a clinical drug candidate for disease modifying therapy in neurodegeneration and a promising scaffold for positron emission tomography tracer design. We used nuclear magnetic resonance spectroscopy and the cryogenic electron microscopy structure of α-synuclein fibrils grown in the presence of lipids to locate anle138b within a cavity formed between two β-strands. We explored and quantified multiple binding modes of the compound in detail using molecular dynamics simulations. Our results reveal stable polar interactions between anle138b and backbone moieties inside the tubular cavity of the fibrils. Such cavities are common in other fibril structures as well.

## Abstract (web summary)

Understanding how small molecules bind to pathological aggregates is of importance for therapeutic and diagnostic development in diseases such as Parkinson’s Disease. Here, the authors reveal a binding site of anle138b to lipid-induced α-synuclein fibrils.

## Introduction

The presence of α-synuclein fibrils in inclusion bodies is a hallmark of neurodegenerative diseases such as Parkinson’s Disease^1^, Multiple System Atrophy^2,3^ and Dementia with Lewy bodies^4,5^. α-Synuclein aggregation is thought to be involved in cellular pathology and spreading between cells^6^. To date, no disease-modifying treatment is available for any of those diseases^7^. Hence, atomic resolution structures of small molecule binding to such pathological protein aggregates are of interest for the development of therapeutics and diagnostics^8,9^. To address this issue, we studied the binding of the small molecule anle138b to α-synuclein fibrils.

Anle138b [3-(1,3-benzodioxol-5-yl)-5-(3-bromophenyl)-1*H*-pyrazole] shows efficacy in animal models for several aggregation related diseases^8,10–14^. It was tested successfully in healthy volunteers^15^ and is currently undergoing phase Ib testing in Parkinson’s disease (NCT04685265). On a molecular level, anle138b was shown to interfere with α-synuclein oligomerization in vitro, inhibiting pore formation in membranes as well as cytochrome *c* leakage from mitochondria and depopulating α-synuclein aggregates in vivo^8,14^. Due to its highly lipophilic nature, it readily crosses the blood-brain-barrier^11^. Its tight binding (*K*_d_ = 190 ± 120 nM) to α-synuclein fibrils^16^ also makes it an attractive starting point for the development of diagnostic positron emission tomography (PET) tracer molecules^9^.

α-Synuclein is a 140 amino-acid intrinsically disordered protein, which has been suggested to participate in synaptic vesicle exocytosis, brain lipid metabolism, and neuronal survival because of its affinity to lipid membranes.^17^ In Parkinson’s disease-related Lewy bodies, α-synuclein fibrils are found to colocalize with lipids, indicating the importance of lipids in the α-synuclein aggregation cascade^18^. We recently studied the aggregation of α-synuclein grown in the presence of negatively charged liposomes consisting of 1-palmitoyl-2-oleoyl-*sn*-glycero-3-phosphate (POPA) and 1-palmitoyl-2-oleoyl-*sn*-glycero-3-phosphocholine (POPC) and observed a segmental folding process^19^. Concordantly, we resolved the resulting protofilament fold L2 of lipid-bound α-synuclein fibrils using cryogenic electron microscopy (cryo-EM)^20^.

## Results

### Anle138b binds to fibril interior

**Fig. 1:**
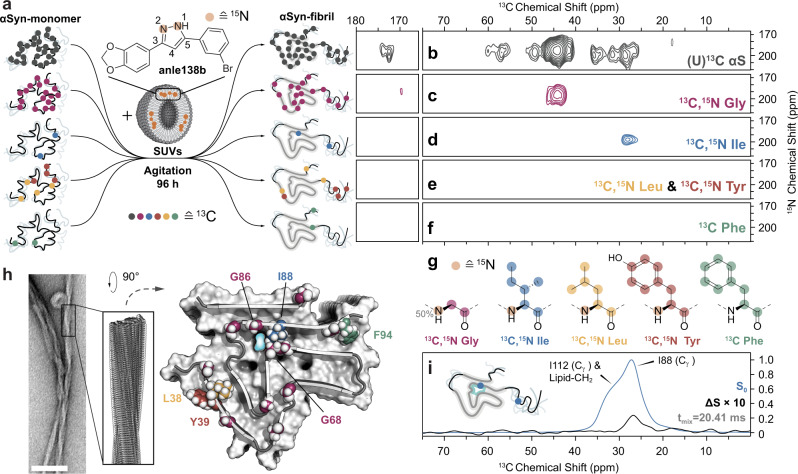
Identification of anle138b binding inside α-synuclein fibrils by DNP-enhanced MAS NMR. **a** Isotope labeling strategy used for the preparation of fibril samples for studies by DNP-enhanced MAS NMR. ^13^C-labeling of amino acids indicated by colored circles: unifom- (gray), Gly- (magenta), Ile- (blue), Leu- (yellow), Tyr- (red), and Phe-labeling (green). ^15^N-labeling on anle138b indicated by orange circles. **b**–**f** Slices of 2D NHHC spectra of α-synuclein fibrils prepared from protein with **b** uniform ^13^C-labeling and **c**–**f** amino-acid-specific isotope labeling on **c** Gly, **d** Ile, **e** Leu and Tyr, and **f** Phe in the presence of 1,2-^15^N-anle138b. The spectral region shown contains only cross-peaks between pyrazole NH of anle138b (195 ppm) and protein carbon atoms. Proton–proton mixing was 200 μs. **g** Chemical structures of amino acids used for specific isotope labeling with ^13^C- labeled nuclei indicated by colored circles (same as in **a**) and ^15^N-labeling indicated by orange circles. **h** Negative stain EM micrograph of fibrils grown in the presence of lipids (scale bar 100 nm) alongside cryo-EM structure of α-synuclein protofilament L2^20^ with color coding of specific isotope-labeled residues. **i** Frequency selective REDOR spectrum (carrier frequency at 26 ppm, Supplementary Table 2) of Ile-labeled α-synuclein fibrils in the presence of 1,2-^15^N-anle138b. The difference spectrum (Δ*S*) is scaled up 10-fold with respect to the reference spectrum (*S*_0_) for clarity. Fibrils were prepared at a lipid to protein to anle138b molar ratio of 10:1:0.5. All spectra were recorded at 600 MHz at 100 K (see Supplementary Table 1) in the presence of TEMTriPol-1.

We located the binding site by isotope labeling anle138b with ^15^N, and directly detecting its interaction with fibrils grown from ^13^C-labeled full-length α-synuclein (Fig. 1a). Such interactions were recorded in two-dimensional (2D) NHHC nuclear magnetic resonance (NMR) spectra with signal enhancement via dynamic nuclear polarization (DNP) and magic-angle spinning (MAS) at 100 K^21^. During fibrillization, we used anle138b doped liposomes consisting of POPA and POPC (1:1). Proton–proton mixing in these experiments results in cross-peaks between nearby nuclei up to a distance of about 6 Å^22^ (Fig. 1b and Supplementary Fig. 1). The most prominent cross-peak at 44.8 ppm is consistent with the pyrazole NH of anle138b binding near C_β_ of Phe, Leu, Tyr, or Ile or C_α_ of Gly. We prepared α-synuclein fibrils with selective ^13^C-labeling on these residues and used 2D ^13^C,^13^C RFDR for the assignment of resonances (Supplementary Fig. 2a, c–f). 2D NHHC spectra of these fibrils in the presence of ^15^N-labeled anle138b (Fig. 1a, b–g, Supplementary Fig. 2b, and Supplementary Table 1) establish Gly as responsible for the peak at 44.8 ppm and Ile as another part of the binding site (28 ppm peak). Interaction with other lipophilic residues, Leu, Tyr, and Phe, or the amine of lysine (Fig. 1e, f and Supplementary Fig. 1b), could not be detected. The interaction of the compound with C_γ_ of an Ile (Fig. 1d) establishes the binding site at I88, since the only other Ile at position 112 can be excluded based on its distinct chemical shift and broad line shape (Supplementary Fig. 2d). The sidechain of I88 is facing towards the protofilament interior and is part of a glycine-rich sequence that encloses a tubular hydrophobic cavity along α-synuclein protofilament L2^20^ (Fig. 1h, PDB ID 8A4L). Rotational echo double resonance (REDOR) experiments confirm close proximity of anle138b to I88 (Fig. 1i). The observed interactions at I88 and Gly, as well as spectroscopic features of G68 and G86 (vide infra), therefore indicate binding of anle138b inside this cavity.

### Bound anle138b changes the local structure

**Fig. 2:**
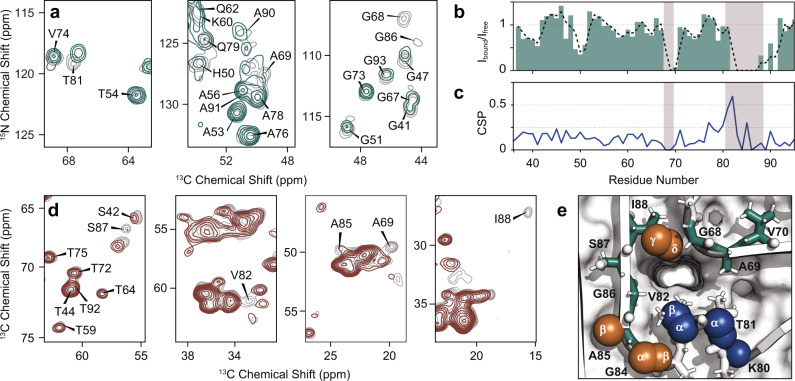
Chemical shift perturbations comparing α-synuclein fibrils grown in the presence and absence of anle138b. **a** Excerpts of carbon-nitrogen projections of 3D (H)CANH spectra of lipidic α-synuclein fibrils. Signal attenuation (*I*_bound_/*I*_free_) and chemical shift perturbation (CSP) are observed comparing spectra of fibrils with anle138b (green, 800 MHz with 55 kHz MAS) and without anle138b (gray, 950 MHz with 100 kHz MAS). **b** Relative peak intensities from 3D (H)CANH spectra and **c**, average CSP from DARR and (H)CANH spectra induced by anle138b inside α-synuclein fibrils plotted per residue. Black dotted line represents a 2-residue moving average; highlighted areas indicate the cavity in protofilament L2. **d** Excerpts of 2D ^13^C,^13^C correlation spectra (20 ms DARR mixing) of α-synuclein fibrils with (red) and without (gray) anle138b acquired at 850 MHz with 17 kHz MAS. **e** Residues enclosing the cavity in α-synuclein protofilament L2 color-coded by nuclei experiencing significant CSP (blue), signal attenuation (green), or both (copper). Source data are provided as a Source Data file.

To probe potential changes in the α-synuclein protofilament structure induced by anle138b, we recorded room temperature MAS NMR spectra. Specifically, we acquired 2D ^13^C,^13^C correlation spectra with dipolar assisted rotational recoupling (DARR) to obtain correlations between carbon atoms, and 3D (H)CANH spectra to obtain correlations between protein backbone C_α_, N_H_ and H_N_. The 2D DARR spectra with long mixing (Supplementary Fig. 3) show similar protein long-range contacts in the presence and absence of anle138b, confirming an overall conserved α-synuclein protofilament fold. The presence of anle138b results in chemical shift perturbation (CSP) for _80_KTV_82,_ A85, and I88 (Fig. 2c and Supplementary Fig. 4a–c) as well as attenuation for residues _67_GGAV_70_ and _82_VEGAGSI_88_ as seen from 3D (H)CANH (Fig. 2a, b and Supplementary Fig. 4d) and 2D DARR spectra (Fig. 2d). This observation indicates increased dynamics at the affected residues compared to the absence of anle138b. Peak-broadening of C_α_ of G68 as well as G86 (Fig. 2a) and particularly C_γ_ of I88 (Fig. 2d) corroborate the contacts observed under DNP conditions. As these residues belong to the two stretches of the α-synuclein sequence which establish the aforementioned cavity in protofilament L2 (Fig. 2e), we conclude that anle138b is embedded within α-synuclein fibrils, while causing only local structural changes.

### Polar contacts govern internal binding

**Fig. 3:**
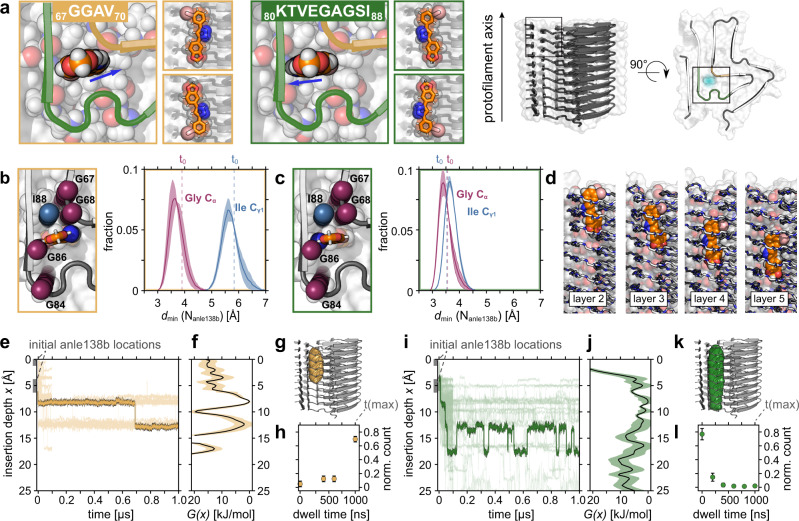
Anle138b binds dynamically inside the α-synuclein protofilament. **a** Starting structures for MD simulations for the four principal poses of anle138b: Pyrazole nitrogen atoms of anle138b (indicated by blue arrows) are oriented either to residues _67_GGAV_70_ (inward-facing, yellow) or to residues _80_KTVEGAGSI_88_ (outward-facing, green) inside the cavity (cyan) of α-synuclein protofilament L2. The bromophenyl moiety can point up or down the protofilament (small panels). A color code denotes the individual simulation sets for each binding pose in all following panels (inward-facing—yellow, outward-facing—green). **b**, **c** Orientation (left) and distributions of minimal distances (right) between nitrogen atoms of anle138b and C_γ1_ of I88 (light-blue), and C_α_ of G67, G68, G84, G86 (magenta) as described in **a**. Dashed lines indicate the initial positions of anle138b at the start of the simulation. **d** Snapshots and **e**, **i** trajectories (representative trajectories as bold lines; others as thin lines for clarity) illustrate discrete translational motion along the protofilament axis and alignment of anle138b with individual β-strand layers. Relative free-energy binding profiles *G(x)* as a function of anle138b insertion depth *x* for combined simulation sets of **f** inward-facing and **j** outward-facing pose. Data are presented as mean values ± SEM (depicted by shading). **g**, **k** Isosurfaces of corresponding anle138b occupancy. **h**, **l** Cumulative histogram of dwell times calculated from anle138b displacements inside the tubular cavity for **h** inward-facing (*n* = 24) and **l** outward-facing (*n* = 218) pose. Data are presented as mean values ± SEM (indicated by error bars). Water and lipid molecules omitted for clarity in all renderings.

**Fig. 4:**
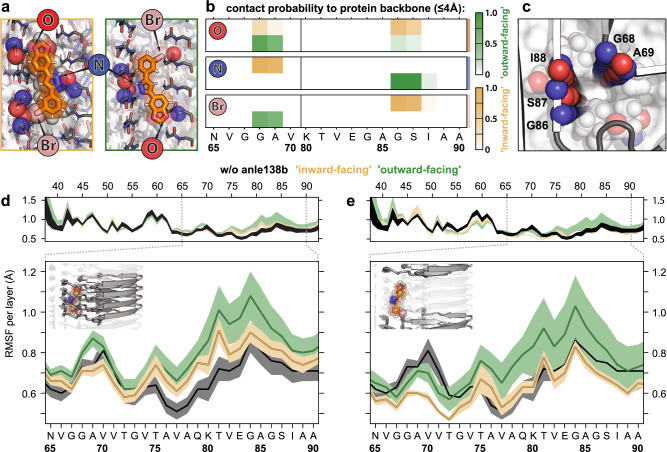
Anle138b forms polar interactions with the protein backbone that alter local structural fluctuations in the protofilament. **a** Polar contacts between anle138b atoms (indicated by arrows) and protein backbone atoms (blue circles—amide nitrogen; red circles—carbonyl oxygen) observed in MD simulations for inward- (yellow) and outward-facing (green) internal binding poses. **b** Contact probabilities for polar interactions with backbone atoms of individual residues for polar interactions defined in **a**. Scale bars (right) indicate contact probabilities. **c** Tubular cavity viewed up close down the long axis of the α-synuclein protofilament (shown without anle138b for clarity). Prominent contact sites in the backbone scaffold are represented by circles (colors as in **a**). **d**, **e** Average root mean squared fluctuations (RMSF) for **d** the four consecutive β-strands in contact with anle138b and **e** β-strands without close polar interactions. Average RMSF without anle138b present shown for comparison (black lines). β-strands of the fibril ends were not considered. Data are presented as mean values ± SEM (depicted by shading). Source data are provided as a Source Data file.

The rather narrow cavity of the α-synuclein protofilament L2 permits four principal internal binding poses for the planar anle138b molecule. Inside the protofilament cavity, the pyrazole nitrogen atoms of anle138b can face either “inward” or “outward” and the bromophenyl ring pointing “up” or “down” with respect to the direction of the long axis of the protofilament (Fig. 3a). Here, the protofilament axis was defined by the direction of the NH vector of every 2^nd^ amino acid in the folded, N-terminal part of the protofilament, starting with the residue G36. All-atom molecular dynamics (MD) simulations (see “Methods” and Supplementary Table 3) were employed to probe if these anle138b-binding poses are indeed consistent with the observations made by NMR. To do so, both tautomers of the anle138b molecule were considered and simulated in the dominant conformer as determined by NMR, respectively (Supplementary Fig. 5). During 40 independent, 1-µs long MD simulations, no unbinding events from the cavity of the α-synuclein protofilament L2 structure were observed independent of the initial binding pose and tautomeric form of anle138b (Supplementary Fig. 6a). Intentionally placing the compound at the edge of the protofilament leads to spontaneous insertion into the internal binding site, demonstrating favorable binding (Supplementary Fig. 7a, b). The steric hindrance imposed by nonpolar side-chain groups (A69, T81, V82, and I88) surrounding the cavity did not allow for rotations of the molecule (Supplementary Fig. 6b). The C_γ_ of I88 showed close and persistent contacts to the anle138b pose with outward-facing pyrazole nitrogen atoms, while the C_α_ of either G67, G68, G84 or G86 did so for both anle138b poses in the simulations (Fig. 3b, c). This is most consistent with NHHC and REDOR spectra for outward-facing anle138b (Fig. 1c, d, i). Since short distances dominate REDOR data, the presence of the inward-facing pose, with larger separation of anle138b nitrogen atoms and I88 C_γ_ is compatible as well. We thus surmise that the outward-facing pose is present in the fibrils, as well as an undetermined amount of inward-facing anle138b. From the MD simulations, we observed a stepwise displacement of anle138b within the internal cavity and along the stacked β-strand layers of the protofilament (Fig. 3d and Supplementary Fig. 6d) on the tens to hundreds of ns timescale (Fig. 3e, h, i, l and Supplementary Movie S1), consistent with the disappearance of some NMR signals at ambient temperatures and the use of cryogenic DNP conditions to localize the compound. Relative free-energy binding profiles (Fig. 3f, g, j, k and Supplementary Fig. 7c) show multiple local energy minima as a function of anle138b insertion depth within the protofilament core for all tested binding poses. However, for inward-facing anle138b binding poses significantly higher energy barriers are found (~20 kJ/mol, Fig. 3f). In concordance with simulations on model systems^23^, anle138b showed characteristic polar interactions with exposed backbone epitopes (Fig. 4a). Polar ladders of backbone atoms lining the cavity (Fig. 4a, c and Supplementary Figs. S6e, S8) interact with anle138b either through hydrogen bonds via the pyrazole ring (outward-facing) or halogen bonds involving the bromine atom (inward-facing) (see “Methods”/Supplementary Information). The common pattern of close contacts between nitrogen atoms of anle138b and protein backbone atoms of G68, A69, or G86 and S87 (Fig. 4b and Supplementary Fig. 6e) agrees with 2D NHHC spectra (Fig. 1c and Supplementary Fig. 1b).

Moreover, when bound inside the protofilament, anle138b molecules markedly alter the structural fluctuations of residues around the cavity during MD simulations. All β-strand layers in proximity to the compound, especially when facing outward, (Fig. 4d) exhibit a higher averaged RMSF for residues _67_GGA_69_, and _80_KTVEGAGSI_88_ consistent with the attenuation and CSP observed by NMR for these perturbed regions (Fig. 2). A similar effect is also seen for layers not in contact with the compound (Fig. 4e).

Simulations with anle138b molecules starting outside the α-synuclein protofilament cavity demonstrate the affinity of anle138b for the lipid environment and pronounced colocalization of the compound around lipid binding sites on the protofilament exterior (Supplementary Figs. S9 and S10). In contrast to the stable internal binding, such binding events are transient, explaining the absence of exterior contacts in NMR spectra (Fig. 1).

## Discussion

Long tubular cavities, as observed in the α-synuclein protofilament L2 and other α-synuclein folds such as PDB IDs 6SSX/6RT0^24^, 7NCI, and 7NCH^25^, (Supplementary Fig. 11) as well as many other amyloid fibrils^26,27^ are a unique feature of the repetitive fibril architecture. This level of commonality across different fibrils could have implications for positron emission tomography radiotracer development. So far, tracer targets are usually identified in exterior binding sites similar to those reported for fibrils of HET-s^28^, amyloid-β (Aβ)^29^, and tau^30,31^. While theoretical studies have predicted the existence of binding sites in the core of fibrillar aggregates^32^, experimental data did not allow for a high-resolution structure^33^. End-to-end filling of tubular cavities in fibrils by hydrophobic molecules has been proposed before^34^. We find selective binding inside and translational motion of anle138b along the α-synuclein fibril cavity, consistent with this model. Our results emphasize that expanding radiotracer target identification to fibril cavities as well as structure-based refinement of cavity binding molecules, such as anle138b, could prove to be beneficial. Fibrils amplified from Parkinson’s Disease patient's brain exhibit a similar fold to the lipidic α-synuclein L2 fibrils and support the relevance of the protofilaments in the present study^35,36^. Further studies are needed to determine whether the internal binding mode of anle138b in lipidic fibrils, is also a prominent feature of anle138b interactions with early aggregates^23^.

We presented a high-resolution structural model for anle138b binding in a lipidic α-synuclein fibril. We explored how the linear arrangement of polar groups inside the continuous tubular cavity formed along the cross-β spine of the protofilament facilitates small molecule binding. It will be of significant interest whether the particular features of glycine enriched tight and hydrophobic cavities as reported here, can also be exploited for selective compound binding to fibrillar aggregates of other amyloidogenic proteins such as Aβ, tau, and prion protein which also bear these cavities.

## Methods

### Preparation of isotope-labeled protein

α-Synuclein was recombinantly produced in *E. coli* strain BL21(DE3) as previously described^37^. Uniformly ^15^N- and ^13^C,^15^N-labeled samples were expressed in a minimal medium supplemented with ^15^NH_4_Cl and ^13^C_6_-d-glucose (Cambridge Isotope Laboratories and Sigma Aldrich). For the production of amino-acid-specific forward-labeled protein (^13^C,^15^N-Leu/Tyr, ^13^C-Phe, ^13^C,^15^N-Ile), the labeled amino acids were added to the minimal medium 1 h before induction of protein expression. For forward labeling with ^13^C- and ^13^C,^15^N-Gly, protein expression was performed with the glycine auxotrophic *E. coli* strain DL39 (DE3) GlyA following a published protocol^38^. The protein was finally dialyzed against buffer (50 mM HEPES, 100 mM NaCl, pH 7.4) to obtain a 0.3 mM solution, and the resulting solution was stored at −80 °C until use.

### Preparation of isotope-labeled anle138b

The general information for the synthesis of compounds was described previously^8^.

### Preparation of [1,2-^15^N_2_]-3-(1,3-benzodioxol-5-yl)-5-(3-bromophenyl)-1*H*-pyrazole



A mixture of [^15^N_2_] hydrazine sulfate (2.57 g, 19.5 mmol), EtOH (20 mL), water (1 mL), and 40% KOH aqueous solution (5.6 g, 40 mmol) was stirred in a sealed pressure flask at 80 °C for 1 h. The mixture was cooled, 3-(1,3-benzodioxol-5-yl)-5-(3-bromophenyl)propane-1,3-dione^8^ (2.43 g, 7 mmol) was added, the reaction vessel was sealed again, and the heterogeneous mixture was stirred at 80 °C for 15 h. The cooled mixture was diluted with water (100 mL), the resulting precipitate was filtered off, washed with water, and air-dried. The crude product was crystallized from *n*-BuOH (10 mL) and vacuum dried to give the title compound as a white solid (1.95 g, 5.65 mmol, 80%). ^1^H NMR (400 MHz, DMSO-d_6_ + 0.5% TFA) *d* = 8.05 (s, 1H), 7.84 (d, *J* = 7.8 Hz, 1H), 7.52 (d, *J* = 8.1 Hz, 1H), 7.45-7.40 (m, 2H), 7.40-7.34 (m, 1H), 7.25 (d, *J* = 3.1 Hz, 1H), 7.01 (d, *J* = 8.1 Hz, 1H), 6.06 (s, 2H). ^13^C NMR (100.6 MHz, DMSO-d_6_ + 0.5% TFA) *d* = 147.9, 147.4, 146.5, 146.3, 133.9, 131.1, 130.6, 127.7, 124.4, 124.2, 122.3, 119.3, 108.8, 105.8, 101.3, 100.1. LC MS (RP18-100Å, gradient 50% CH_3_CN/50% H_2_O to 100% CH_3_CN in 30 min), RT 14.2 min and mass 345.1 (100%), 347.2 (98%), [M + H]^+^. TLC (SiO_2_, *n*-hexane/EtOAc = 2/1) R_f_ 0.39, m.p. 195–197 °C.

### Preparation of [3,4-^13^C_2_]-3-(1,3-benzodioxol-5-yl)-5-(3-bromophenyl)-1*H*-pyrazole



#### [1,2-^13^C_2_]-1-(1,3-benzodioxol-5-yl)ethanone

Starting material [1,2-^13^C_2_]-1-(1,3-benzodioxol-5-yl)ethanone was synthesized by Friedel-Crafts reaction of 1,3-benzodioxole and [1,2-^13^C_2_]-acetic acid (99% ^13^C) according to procedure described in^39^. In an atmosphere of nitrogen, triflic anhydride (5.0 g, 17.7 mmol) was added to [1,2-^13^C_2_]-acetic acid (99% ^13^C) (1 g, 16.1 mmol) with cooling (10 °C bath temperature). After stirring at room temperature for 5 min, 1,3-benzodioxole (1.97 g, 16.1 mmol) was added, and the stirring at room temperature was continued for 30 min. The mixture was quenched with saturated NaHSO_4_ solution (30 mL) and extracted with EtOAc (30 mL). The separated organic layer was washed with water, brine, dried over Na_2_SO_4_, and concentrated under reduced pressure. The residue was purified by flash chromatography on silica gel (*n*-hexane/EtOAc = 3/1) to afford the title compound (1.63 g, 9.8 mmol, 61%) as a white solid. ^1^H NMR (400 MHz, CDCl_3_) *d* = 7.55 (ddd, *J* = 8.1, 4.1 1.7 Hz, 1H), 7.44 (dd, *J* = 3.6, 1.7 Hz, 1H), 6.85 (d, *J* = 8.1 Hz, 1H), 6.04 (s, 2H), 2.54 (dd, *J* = 127.5, 5.9 Hz, 3H). ^13^C NMR (100.6 MHz, CDCl_3_) *d* = 196.2 (d, *J* = 43.1 Hz, ^13^C-enriched), 151.7, 148.2, 132.1 (dd, *J* = 53.9, 14.1 Hz, ^13^C-enriched), 124.7, 108.0, 107.8, 101.8, 26.4 (d, *J* = 43.1 Hz, ^13^C-enriched). LC MS (RP18-100Å, gradient 50% CH_3_CN/50% H_2_O to 100% CH_3_CN in 30 min), RT 6.2 min and mass 166.9 [M + H]^+^. TLC (SiO_2_, *n*-hexane/EtOAc = 3/1) R_f_ 0.5, m.p. 87–89 °C.

#### [3,4-^13^C_2_]-3-(1,3-benzodioxol-5-yl)-5-(3-bromophenyl)-1*H*-pyrazole

The compound was synthesized according to the procedure described in^8^. To a solution of [1,2-^13^C_2_]-1-(1,3-benzodioxol-5-yl)ethanone (830 mg, 5.0 mmol) and methyl 3-bromobenzoate (1.35 g, 6.3 mmol) in DMSO (12 mL) and THF (3 mL) was added sodium hydride (60% in oil, 6.3 mmol, 252 mg). The reaction mixture was stirred at 20 °C for 24 h, then poured into 60 mL of ice water containing 85% H_3_PO_4_ (0.6 mL) and stirred for 1 h. The resulting precipitate was filtered off, washed with water (3.10 mL), MeOH (5 mL), hexane (10 mL), and air-dried to give the crude intermediate [1,2-^13^C_2_]-3-(1,3-benzodioxol-5-yl)-5-(3-bromophenyl)propane-1,3-dione (1.57 g) as a yellow solid which was used for the next step without purification. To a suspension of this in EtOH (20 mL) hydrazine hydrate (500 mg, 10 mmol) was added. The reaction mixture was stirred at 78 °C for 15 h, cooled down, and concentrated in vacuum. The crude product was crystallized from *n*-BuOH (10 mL) and vacuum dried to give 1.12 g of the title compound as a white solid (3.2 mmol, 65% over two steps). ^1^H NMR (400 MHz, DMSO-d_6_ + 0.5% TFA) *d* = 8.05 (s, 1H), 7.85 (d, *J* = 7.7 Hz, 1H), 7.52 (d, *J* = 8.1 Hz, 1H), 7.35–7.41 (m, 3H), 7.26 (dd, *J* = 176.3, 6.2 Hz, 1H, ^13^C-enriched), 7.01 (d, *J* = 8.1 Hz, 1H), 6.07 (s, 2H). ^13^C NMR (100.6 MHz, DMSO-d_6_ + 0.5% TFA) *d* = 147.9 (d, *J* = 5.8 Hz, ^13^C-enriched), 147.4, 146.6 (d, *J* = 61.0 Hz, ^13^C-enriched), 146.3 (d, *J* = 62.7 Hz, ^13^C-enriched), 133.8, 131.1, 130.7, 127.7, 124.4 (d, *J* = 62.5 Hz, ^13^C-enriched), 124.2, 122.3, 119.3, 108.9 (d, *J* = 3.9 Hz, ^13^C-enriched), 105.8, 101.3, 100.1 (d, *J* = 62.7 Hz, ^13^C-enriched). LC MS (RP18-100Å, gradient 50% CH_3_CN /50% H_2_O to 100% CH_3_CN in 30 min), RT 14.2 min and mass 345.1 (100%), 347.1 (98%), [M + H]^+^. TLC (SiO_2_, *n*-hexane/EtOAc = 2/1) R_f_ 0.39, m.p. 195–197 °C.

### Preparation of α-synuclein fibrils

Samples of α-synuclein fibrils were prepared as previously reported^19^. In brief, vesicles were prepared by mixing 1-palmitoyl-2-oleoyl-*sn*-glycero-3-phosphocholine (POPC), 1-palmitoyl-2-oleoyl-*sn*-glycero-3-phosphate (POPA), sodium salt and anle138b each dissolved in chloroform respectively and evaporating the solvent under a N_2_-stream and lyophilized overnight. SUVs were obtained by repeated sonication of a solution of 1.5 mM POPC, 1.5 mM POPA, and 150 μM anle138b. Vesicles were incubated with 70 μM α-synuclein in buffer (50 mM HEPES, 100 mM NaCl, pH 7.4) at a lipid to protein ratio of 10:1 and a compound to protein ratio of 1:2 and subjected to repeated cycles of 30-s sonication (20 kHz) at 37 °C followed by an incubation period of 30 min. After 96 h, fibrils were harvested by centrifuging at 152,460 × *g* (TLA-100.3 rotor in an Optima™ MAX-TL) for 1 h at 4 °C. The supernatant was removed, and the resulting pellet was washed with fresh buffer and centrifuged again 212,940 × *g* for 10 min at 18 °C. Excess moisture was removed, and the pellet was immediately packed into rotors for ssNMR.

### ssNMR spectroscopy

For ssNMR studies, fibrils were grown from (U)-^13^C^15^N-α-synuclein in the presence of vesicles of POPA and POPC (1:1) containing 3,4-^13^C-anle138b at a L/P ratio of 5:1. For room temperature experiments, both samples with and without the compound were prepared at the same time and a single sample each was used for all 3D (H)CANH and 2D ^13^C^13^C-DARR experiments. Fibrils were packed into ssNMR rotors by cutting off the bottom of the tube and centrifuging the pellet directly into the rotor of choice through a custom-made filling device made from a truncated pipette tip. Last, the sample was centrifuged into the rotor in an ultracentrifuge packing device for 30 min at 98,381×*g* in a SW 32 Ti rotor in an Optima™ L-80 XP Ultracentrifuge (both Beckman Coulter)^40^.

3D (H)CANH experiments^41^ on fibrils in the presence of anle138b were recorded on an 800 MHz Bruker Avance III HD spectrometer at a magnetic field of 18.8 T equipped with a 1.3-mm magic-angle spinning (MAS) HCN probe and MAS at 55 kHz. The temperature of the cooling gas was set to 250 K, resulting in an estimated sample temperature of 20 °C.

3D (H)CANH spectra on fibrils in the absence of anle138b were recorded on a 950 MHz Bruker Avance III HD spectrometer at a magnetic field of 22.3 T equipped with a 0.7 mm HCDN probe and MAS at 100 kHz. The temperature of the cooling gas was set to 250 K, resulting in an estimated sample temperature of 20 °C.

2D ^13^C,^13^C-DARR spectra were acquired on an 850 MHz Avance III spectrometer with a 3.2-mm MAS HCN probe with mixing times of 200 ms at a magnetic field of 20.0 T and MAS at 17 kHz. The temperature of the cooling gas was set to 265 K, resulting in an estimated sample temperature of 20 °C.

3D (H)CANH spectra were acquired in three blocks of 32 h in the presence and four blocks of 19 h in the absence of anle138b. All 2D ^13^C,^13^C DARR spectra were acquired in 34 blocks of 7.2 h in the presence and 25 equivalent blocks in the presence of anle138b. All related blocks were corrected for linear drift of the static magnetic field using an in-house program executed from the command line in Bruker Topspin 4.0.8^42^. The drift-corrected blocks were then averaged and processed as one spectrum. Spectra were analyzed using CcpNmr Analysis and NMRFAM-Sparky^43^. Peak intensities were determined by integrating cross-peaks using the Lorentzian fit algorithm in Sparky, not allowing peak center motion. Assignments on α-synuclein fibrils in the absence of anle138b had previously been reported^19^. Chemical shift perturbations were calculated as the average of H_N_, N_H_, C_α_, and C_β_ chemical shifts.

### DNP-enhanced ssNMR

For DNP-enhanced ssNMR, fibrils were grown as for ssNMR measurements, but with isotopic labeling (see the section “Preparation of isotope-labeled protein”) as indicated in the text.

TEMTriPol-1^44^ was synthesized based on published protocols^45^. A solution of ^13^C-depleted *d*_*8*_-Glycerol (70%-vol in water), containing 4–5 mM TEMTriPol was added to the resulting aggregate-lipid mixture and mixed thoroughly. Samples were packed into 3.2 mm zirconia ssNMR rotors via a custom-made filling device made from a truncated pipette tip. Finally, the sample was centrifuged into the rotor in an ultracentrifuge packing device for 30 min at 98,381 × *g* in a SW 32 Ti rotor in an Optima™ L-80 XP Ultracentrifuge.

All DNP spectra were recorded on a 600 MHz Bruker Avance III HD spectrometer at a magnetic field of 14.1 T equipped with a 3.2 mm low temperature (LT) HCN MAS probe. MAS frequencies are indicated in Supplementary Table 1. For DNP radical-proton transfers, 395 GHz microwave irradiation from a gyrotron oscillator was delivered to the sample through a corrugated waveguide. Samples were cooled with a second-generation BRUKER liquid nitrogen cold cabinet, operating at approx. 100 K.

The DNP ssNMR experiments were performed with a recycle delay of 2–4 s (1.2 × T_1_) for each sample and radio-frequency power levels of 100, 72, and 42 kHz for ^1^H, ^13^C, and ^15^N, respectively. During acquisition, during REDOR, as well as during radiofrequency driven recoupling (RFDR), SPINAL64^46^ decoupling was used with 100 kHz power on ^1^H.

2D ^13^C,^13^C spectrum, 1024 increments were acquired using 1.3 ms RFDR mixing using an xy-8 phase cycling and 83 kHz pi-pulses. ^1^H,^13^C cross-polarization (CP) was applied for 1.5 ms with a 90–100% ramp on the proton channel.

For NHHC spectra, 32 increments were acquired with 200 μs of proton–proton mixing (Supplementary Table 1). The ^1^H,^13^C CP was set to 300 μs to limit transfers to the direct proton environment, and ^1^H,^15^N CP was applied for 800 μs with an 80–100% ramp on the proton channel.

Frequency selective ^13^C,^15^N REDOR experiments^47^ were performed with 180° equivalent Q3 shape pulses on both ^13^C and ^15^N concurrently (Supplementary Table 2). The total time of the experiment was 4.67 h with a recycle delay of 2 s (4096 Scans).

### Solution NMR spectroscopy

For determination of the conformation of anle138b we prepared a 20 mM solution of vesicles consisting of POPC and POPA (1:1) containing 1 mM anle138b in buffer (50 mM HEPES, 100 mM NaCl, pH 7.4) with 10% D_2_O and 100 μM 2,2-dimethyl-2-silapentane-5-sulfonate sodium salt (IUPAC: 3-(trimethylsilyl)propane-1-sulfonate, sodium salt). NOESY spectra were acquired with 72 scans using 256 increments in the indirect dimension and a relaxation delay of 2.3 s. The mixing time was set to 3 ms. Proton 90° flip pulses were 14.4 μs. Water suppression was achieved by presaturation at a power level of 100 Hz. Experiments were recorded on a Bruker 900-MHz spectrometer. The temperature during measurements was kept at 298 K. 2D datasets were processed in NMRPipe^48^ and analyzed in NMRFAM-Sparky^43^. Spectral traces were fitted to a mixed Gaussian and Lorentzian function using the line shapes tool in Bruker Topspin 4.0.8.

### Molecular dynamics (MD) simulations

The GROMACS 2018.3 simulation software package^49,50^ was used to set up and carry out the MD simulations. For all simulations, the AMBER99SB*-ILDN^51–53^ force field and the ion parameters in ref. 54 for use with the TIP3P water model^55^ were employed. A modified version^56^ of the all-atom Slipids force field^57–59^ was used for the description of POPC lipids. The parameterization scheme for the small molecule compound anle138b is detailed elsewhere^23^. In order to speed up the simulations, aliphatic hydrogen atoms were converted to virtual sites^60^ and bonds between all atoms in protein, lipid, and ligand molecules were constrained using the P-LINCS^61^ algorithm. Water molecules were constrained using SETTLE^62^. The integration timestep was set to 4 fs. Neighbor lists were updated with the Verlet list scheme^50,63^. The long-range electrostatic interactions were treated using the Particle Mesh Ewald (PME) method^64,65^, with a 1.0 nm real-space cutoff. The van der Waals interactions were cut off at 1.0 nm. A dispersion correction for energy and pressure was applied. For production runs, the temperature was kept at 300 K by applying the velocity-rescaling^66^ algorithm. The pressure was held constant at 1 bar by using the Parrinello–Rahman barostat^67^. All simulations were carried out using periodic boundary conditions for the simulation box.

#### Setup of α-synuclein protofilament structure models

Simulations of α-synuclein β-sheet constructs were carried out based on structure coordinates from the α-synuclein fibril polymorph L2A (PDB ID 8A4L; EMDB ID EMD-15148)^20^ constituted by the residues 33-96, and the resolved parts of the N-terminus (residues 14-24). For simplicity, we refer to the protofilament structure of polymorph L2A as L2, since it is structurally identical to the co-occurring polymorph L2B. The simulation model (see Supplementary Table 2) consisted of one protofilament structure with ten β-strand layers stacked on top of each other, respectively. A rhombic dodecahedron box was used for all simulations with the distance between the solute and the box edge of at least 2.5 nm. The resulting box size dimensions converged to ~12.8 × 12.8 × 12.8 nm^3^ after initial equilibration prior to starting the production runs. For each simulation system, the titratable groups of the protein structure were protonated according to their standard protonation states at pH 7, and the N- and C-termini of the peptides were capped with acetyl and N-methyl groups, respectively. Counterions (Na^+^, Cl^−^) were added to yield an ionic strength of 150 mM and to neutralize the net system charge. In all simulations, 100 POPC molecules were present and initially placed randomly in the solvent.

In total, the simulation systems contained ~155,000 atoms, including ~41,000 water molecules and 316 ions.

#### The MD production runs

All production runs were preceded by a multi-step energy minimization and equilibration of the simulation systems. Initial velocities were taken according to the Maxwell-Boltzmann distribution at 300 K. Simulations were carried out in the presence and absence of anle138b. Two principal conditions were chosen for simulations with α-synuclein protofilament models and anle138b:

A. One anle138b molecule was modeled inside the internal cavity such that its long axis aligned with the protofilament axis, and the solvent-facing atoms of the compound aligned with the surface of the protofilament tip. The compound was inserted without interatomic overlaps to the side-chain atoms of the cavity enclosing residues, in a total of four different poses: with the pyrazole nitrogens oriented toward residues _67_GGAV_70_ (inward-facing) or residues _81_TVEGAGSI_88_ (outward-facing), and with an up or down positioning of the bromophenyl moiety of anle138b with respect to direction of the closest edge of the protofilament. Each initial pose was energy minimized and subsequently equilibrated for 1 ns with position restraints acting on the protein-heavy atoms.
B. Ten anle138b molecules were placed randomly in the solvent and at least 1 nm away from any protein-heavy atom.

The total simulation time for this study adds up to 100 μs, resulting in 50 μs sampling of the internally bound state of anle138b. From the individual simulation trajectories, snapshots were collected every 100 ps. Only the last 75% of the trajectory data were used for subsequent analyses as a measure to discard the initial equilibration phase and structural relaxation of the systems.

#### Analysis and visualization

The MD simulation trajectories were preprocessed and analyzed by tools provided by the GROMACS 2018.3 simulation software package^49,50^, as well as custom-made bash, awk, and python scripts. Renderings of atomic coordinates were carried out with the PyMOL molecular visualization system^68^. Graphs and figures were created using gnuplot 5.2 and inkscape 1.1.

#### Relative free-energy binding profile

For each simulation frame, the center of mass distance between the pyrazole ring of the anle138b molecule and the protofilament tip (edge β-strand layer) was determined (see e.g., Fig. 3e). The distances from each simulation trajectory set were combined and sorted into 1-d bins. The logarithm of the probability *p* = *x*_*i,j*_*/x*_*total*_ was calculated for each bin *i*, *j*, where *x*_i,j_ is the number of frames in the bin and *x*_total_ is the total number of frames. Multiplying a constant factor (-RT, with *T* = 300 K) to *p(x)* results in *G(x)* in units of kJ/mol.

#### Contact analysis

The frequency of short-range interatomic interactions between the anle138b small molecules, POPC lipids, and the peptide aggregates were quantified with the g_contacts program^69^. An anle138b or POPC contact was considered as formed, if any heavy atom of the respective molecules was within a cutoff of 0.4 nm from any heavy atom of a protein residue. In addition, and according to the isotope labeling scheme in the NMR experiments, if the 1,2-N atoms of the anle138b molecule were found within a cutoff of 0.5 nm from any backbone carbon or nitrogen atom of a protein residue, a contact was considered formed. Contacts were averaged over time and over the individual trajectories of each simulation set. If not explicitly stated, only contacts to the core β-strand layers (neglecting the top β-strands on both protofilament edges) were considered for analysis. Hydrogen bond (HB) energies were calculated according to the empirical formula provided in ref. 70. For the definition of halogen bonds (XB) the following criteria were adopted from ref. 71: (a) distance between halogen atom and carbonyl oxygens ≤ 0.36 nm; (b) angle between XB and halogen–carbon bond ≥ 150°.

#### Anle138b-binding mode classification

Based on the previously described heuristic classification of anle138b interaction patterns to oligomeric forms of peptide aggregates in terms of atomic contact numbers and the presence of hydrogen bonds^23^, the following binding modes to fibrillar aggregates were defined: (I) “unbound”, no atomic contacts; (II) “partially bound”, fewer than half of the anle138b atoms in contact with peptide heavy atoms, mostly describing transient binding events; (III) “no HB or XB”, half or more of the anle138b atoms in contact with peptide heavy atoms, no HB or XB interactions to the backbone or side-chain atoms; (IV) “polar interactions”, at least one HB or XB between anle138b and the protofilament structure; (IVa) “pyrazole HB”, at least one HB between anle138b (via the pyrazole ring) and the protofilament structure; (IVb) “benzodioxole HB”, at least one HB between anle138b (via the benzodioxole ring) and the protofilament structure; (IVc) “bromophenyl XB”, at least one XB between anle138b (via the bromophenyl ring) and the protofilament structure.

### Reporting summary

Further information on research design is available in the Nature Research Reporting Summary linked to this article.

## Supplementary information

Supplementary Information

Description of additional supplementary files

Supplementary Movie 1

Reporting Summary

## Data Availability

NMR spectra raw data generated in this study, Negative stain EM micrographs for αSyn fibrils in the presence of anle138b, and mass spectrometry data for the synthesis of selectively labeled α-synuclein and ^13^C- and ^15^N-labeled anle138b, respectively, have been deposited in the open research data repository Edmond at [10.17617/3.9C6TEW]. The molecular dynamics data generated in this study have been deposited in the Edmond data repository at [10.17617/3.R4FJQG]. Assigned chemical shift data (H_N_, C_α_, C_β_, and C′) for α-synuclein fibrils were deposited in the BMRB as updates under the accession number 50585. Atomic coordinates for L2A-fibrils of αSyn used for MD simulations and visualization in this manuscript are available in the Protein Data Bank under the accession code 8A4L. Previously published structures of αSyn fibrils used for a comparison of tubular fibril cavities are available in the Protein Data Bank under the accession codes 6SSX, 7NCI, and 7NCH. All other data generated or analyzed during this study are included in this published article (and its supplementary information files). Source data are provided with this paper.

## Source data

Source Data
