# Supplementary material for: The clinical drug candidate anle138b binds in a cavity of lipidic α-synuclein fibrils
Source: Nat Commun. 2022 Sep 14;13:5385. doi: 10.1038/s41467-022-32797-w (PMC9474542; doi:10.1038/s41467-022-32797-w)
Supplement: Supplementary file 1 — Supplementary Information [file 41467_2022_32797_MOESM1_ESM.pdf]

Supplementary Information

The clinical drug candidate anle138b binds in a cavity of lipidic α-synuclein fibrils

Authors: Leif Antonschmidt[1*], Dirk Matthes[2*], Rıza Dervişoğlu[1*]†, Benedikt Frieg[3], Christian Dienemann[4], Andrei Leonov[1,5], Evgeny Nimerovsky[1], Vrinda Sant[1], Sergey Ryazanov[1,5], Armin Giese[5,6], Gunnar Schröder[3], Stefan Becker[1], Bert L. de Groot[2#], Christian Griesinger[1,7#], Loren B. Andreas[1#]

Affiliations:

[1]NMR-based Structural Biology, Max Planck Institute for Multidisciplinary Sciences, Göttingen, Germany.

[2]Department of Theoretical and Computational Biophysics, Max Planck Institute for Multidisciplinary Sciences, Göttingen, Germany.

[3]Institute of Biological Information Processing (IBI-7: Structural Biochemistry), Forschungszentrum Jülich; Jülich, Germany.

[4]Department of Molecular Biology, Max Planck Institute for Multidisciplinary Sciences, Göttingen, Germany.

[5]MODAG GmbH, Mikroforum Ring 3, 55234 Wendelsheim, Germany.

[6]Center for Neuropathology and Prion Research, Ludwig-Maximilians-University Munich, Munich, Germany

[7]Cluster of Excellence "Multiscale Bioimaging: From Molecular Machines to Networks of Excitable Cells" (MBExC), University of Göttingen, Göttingen, Germany.

†Present address: Max Planck Institute for Chemical Energy Conversion, Mülheim an der Ruhr, Germany.

# Correspondence and requests for materials should be addressed to bgroot@gwdg.de, cigr@mpinat.mpg.de or land@mpinat.mpg.de
* These authors contributed equally.

Contents

Supplementary Figures 1 - 11 - pages 2 - 13

Supplementary Tables 1 - 3 - pages 14 - 16

Supplementary Figures

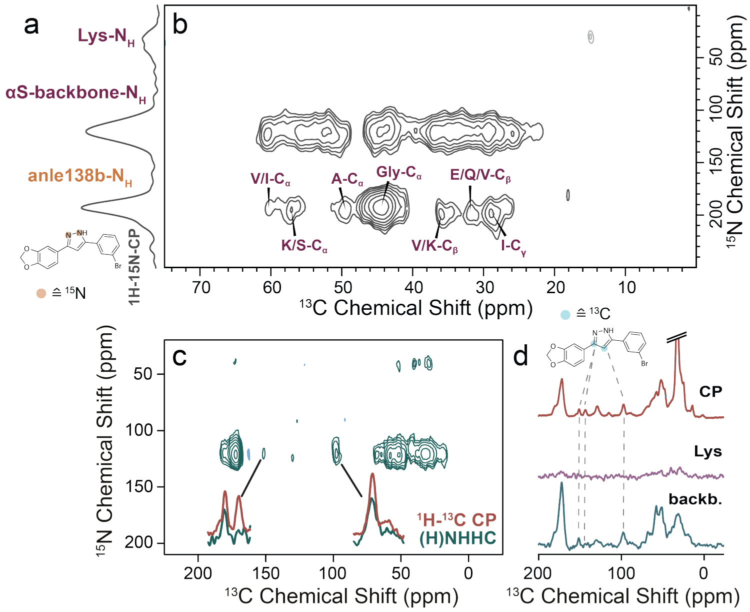

Supplementary Fig. 1. 2D (H)N(HH)C spectra of α-synuclein fibrils in the presence of anle138b. a ^{1}H-^{15}N-CP spectrum and **b** aliphatic region of a 2D (H)N(HH)C spectrum acquired on uniformly ^{13}C-labelled α-synuclein-fibrils in the presence of vesicles of POPA/POPC (1:1) at a L/P ratio of 10:1 containing 0.5 eq. ^{15}N-labelled anle138b. Intramolecular cross-peaks for protein resonances arise from natural abundance ^{15}N-nuclei. Peaks are labelled by potential residue types giving rise to the observed interactions with anle138b. **c** 2D (H)N(HH)C spectrum of ^{15}N-labelled α-synuclein fibrils in the presence of 3,4-^{13}C-anle138b. Magnified cross sections show comparison of signals stemming from anle138b to the corresponding signals in a ^{1}H-^{13}C-CP spectrum. **d** Cross-sections of the 2D NHHC spectrum in **c** at 30 ppm (red, Lys sidechain nitrogen) and 120 ppm (green, backbone nitrogen) compared to ^{1}H-^{13}C-CP spectrum (blue). All spectra were recorded at 600 MHz with 12.5 kHz MAS at 100 K and in the presence of TEMTriPol-1.

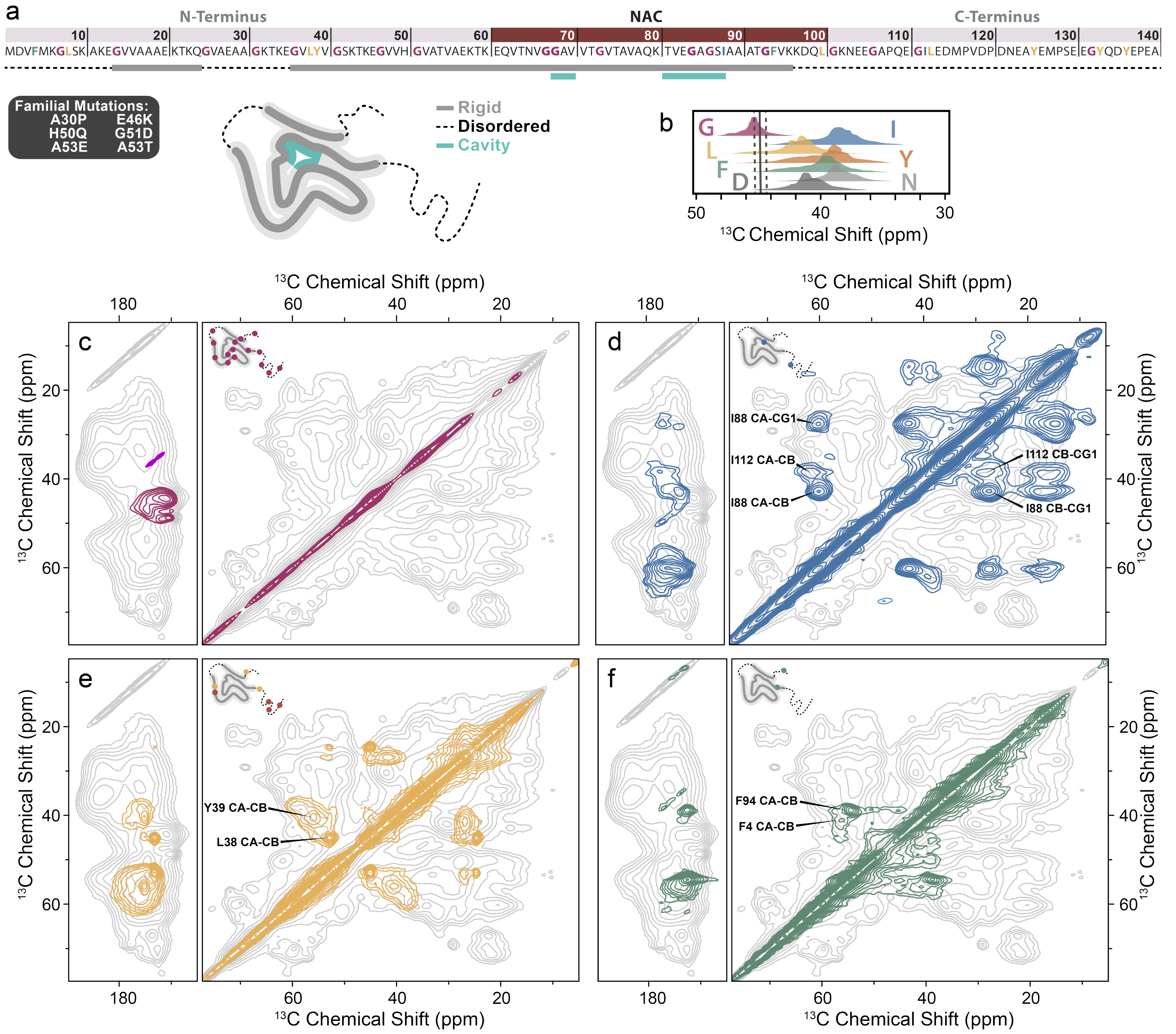

Supplementary Fig. 2. RFDR spectra of selectively isotope labelled α-synuclein fibrils studied by DNP. a α-synuclein sequence with highlighting residues which have been selectively labelled for interaction studies by 2D NHHC. Bar indicates rigid residues in the protofilament core, dotted line indicates disordered residues. Cyan bars binding sites identified from combined ssNMR data. **b** Statistical distributions of C_α (Gly) and C_β (I, L, F, Y, N, D) chemical shifts obtained from the Biological Magnetic Resonance Data Bank (BMRB). Solid line indicates chemical shift of most prominent cross-peak between anle138b and α-synuclein in Fig. 1B (dotted lines indicate expectable temperature shift at 100K). (C-F) 2D ^{13}C^{13}C correlation spectra (2.6 ms RFDR mixing) of fibrils prepared with **c** ^{13}C^{15}N-Gly, **d** ^{13}C^{15}N-Ile, **e** ^{13}C^{15}N-Leu (yellow) and ^{13}C^{15}N-Tyr (orange) and **f** ^{13}C^{15}N-Phe in the presence of POPA and POPC (1:1) at a L/P ratio of 10:1 and 0.5 eq. ^{15}N-labelled anle138b. Spectra show peaks for residues of both the rigid fibril core (residues 38 to 95, labelled in spectra), such as L38, Y39, G68, I88, F94 as well as residues from the disordered N terminus, such as F4, G7 and L8 and C terminus such as I112, L113, Y124, and G132. Spectra were recorded at 100 K, resulting in freezing of disordered parts of the protein, leading to observation of broad peaks because of conformational heterogeneity. C- and N-terminal residues, disordered and flexible and hence not observable in ssNMR spectra at room temperature, are observed as broad peaks at 100 K, because heterogeneous conformations are frozen out. All spectra are overlayed with spectra of uniformly ^{13}C-labelled α-synuclein fibrils. Spectra were recorded at 600 MHz with 12.5 kHz MAS in the presence of TEMTriPOL-1.

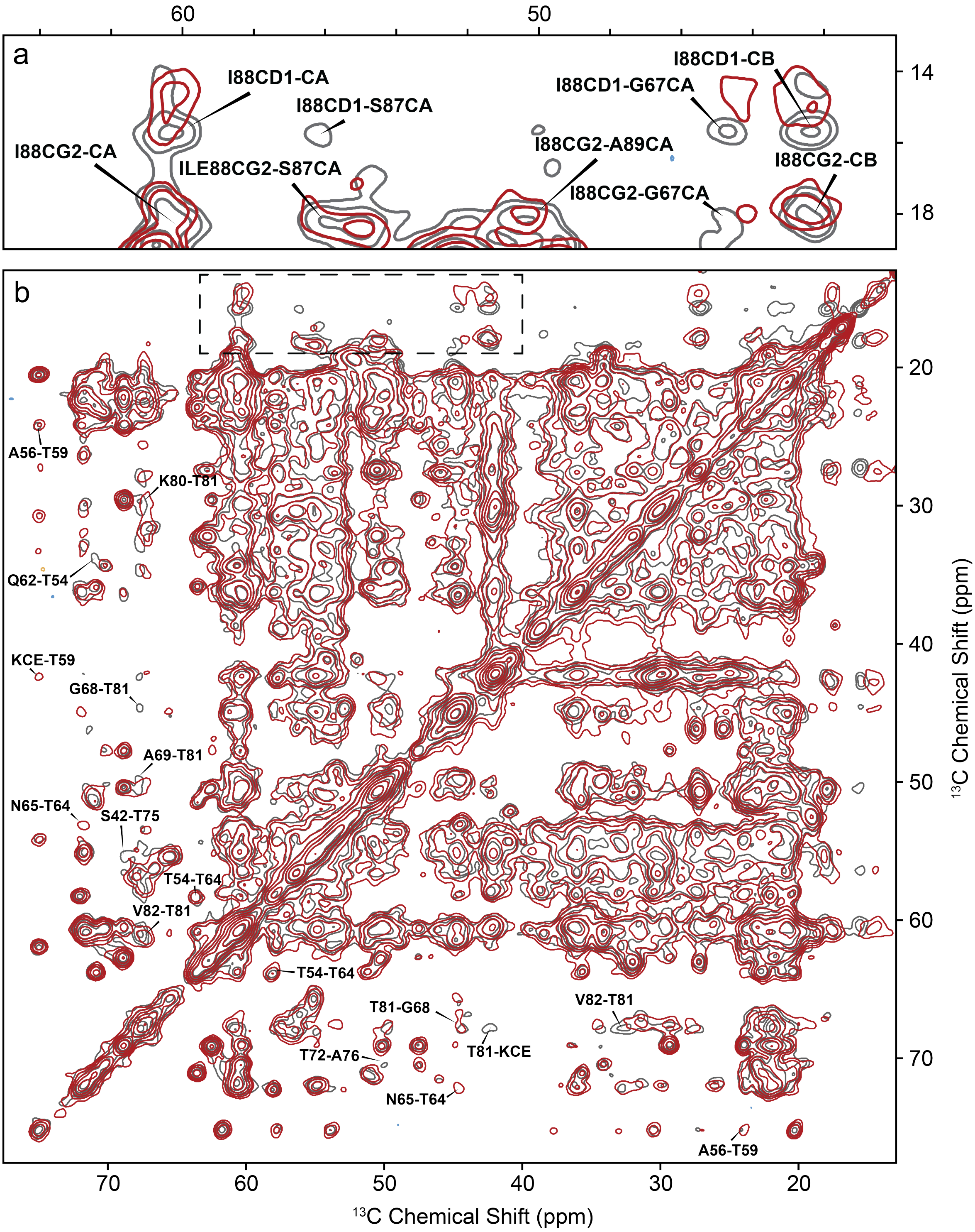

Supplementary Fig. 3. 2D ^{13}C-^{13}C-DARR for confirmation of α-synuclein fibril fold. a Excerpt of the 2D ^{13}C-^{13}C DARR (full spectrum in **b**) spectrum (200 ms mixing time) acquired on ^{13}C^{15}N-α-synuclein fibrils in the presence (red/yellow) and absence (grey/blue) of anle138b, showing cross-peaks originating from I88. Cross peaks between I88 and G67, which confirm the conserved protein fold, are broadened due to increased dynamics induced by anle138b but are generally retained. **b** Full aliphatic region 2D ^{13}C-^{13}C DARR spectrum. Labels indicate cross-peaks corresponding to long distance contacts (i → i±n ; n≥3) and/or peaks which are influenced by the presence of anle138b. The grey dotted box indicates the area magnified in **a**. Fibrils were grown in the presence of POPA and POPC at a Lipid to Protein ration of 5:1.

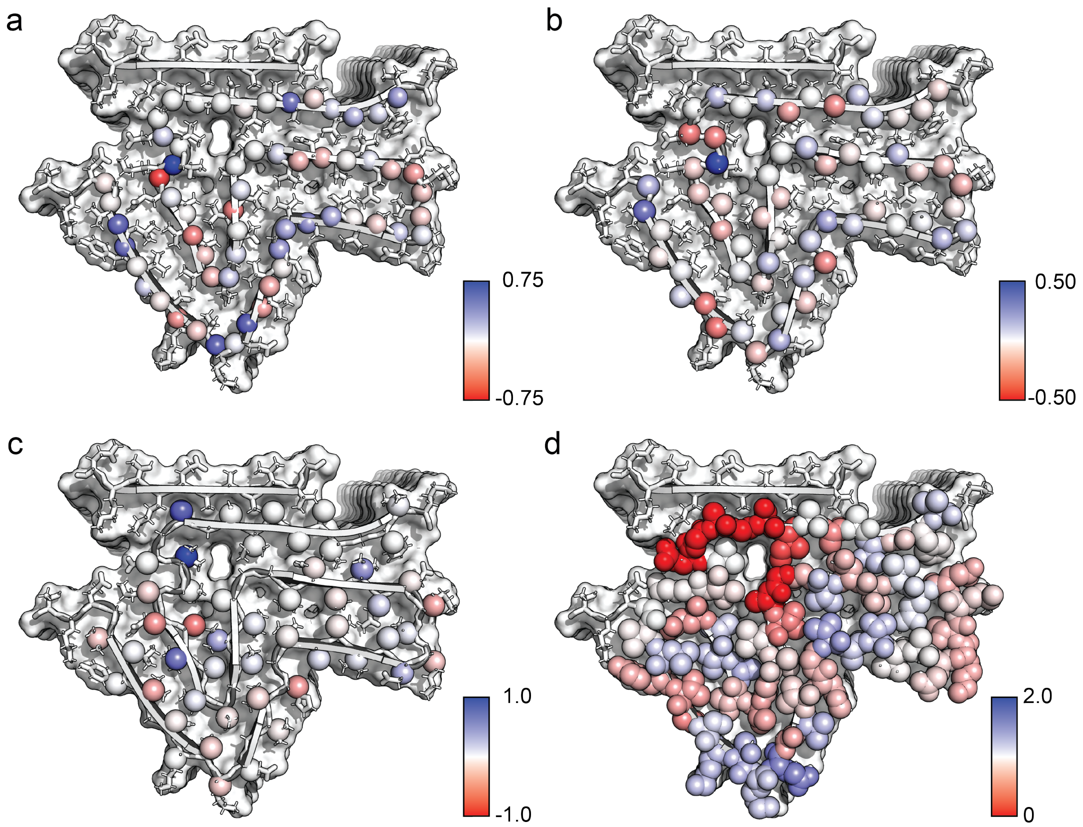

Supplementary Fig. 4. Intensity and chemical shift changes induced by anle138b. Chemical shift changes (δ_{bound}-δ_{free}) for **a** N_H-, **b** C_α- and **c** C_β-atoms as well as **d** ratios of integrated peak intensities (I_{bound}/I_{free}) from 3D (H)CANH spectra observed in the presence of anle138b. All values are plotted onto the structure of α-synuclein protofilament L2 (PDB ID 8A4L). Source data are provided as a Source Data file.

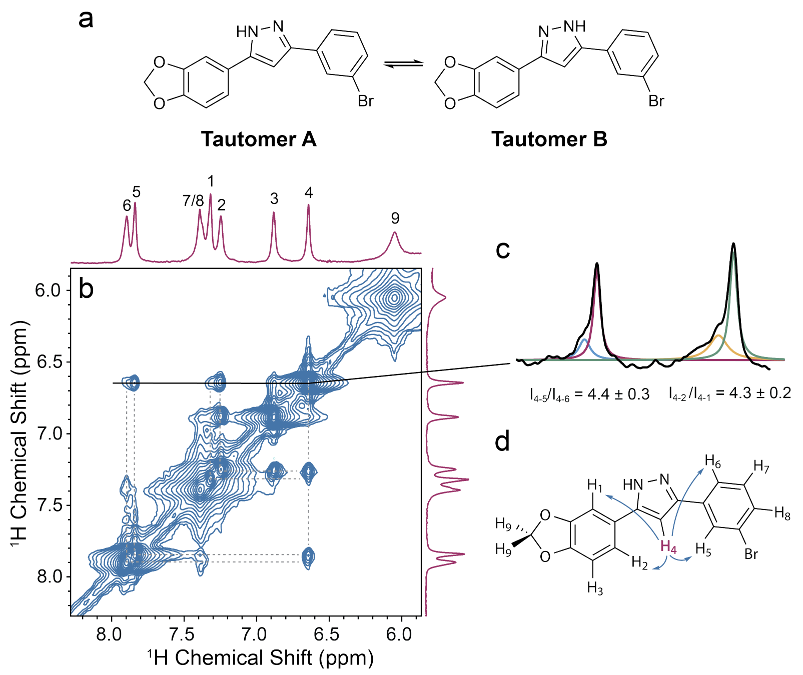

Supplementary Fig. 5. Conformer of anle138b. a Chemical structures of the two tautomeric forms of anle138b. **b** 2D ^{1}H-^{1}H NOESY spectrum (mixing time τ_m = 3 ms) of anle138b inside SUVs consisting of POPA and POPC (1:1). Spectrum was acquired at 900 MHz at RT. **c** 1D slice through cross-peaks in **b** originating from interaction of the pyrazole proton H_4 to its neighboring protons. Ratios of cross-peak intensities obtained from fitting a mixed gaussian and Lorentzian function indicate close proximity to protons H_2 in the benzodioxole moiety and H_5 in the bromophenyl moiety. **d** Chemical structure of anle138b with indication of the NOE transfer pathways leading to the cross-peaks in **c**. Differential proximities towards the neighboring ring protons originate from restricted ring motion in the lipid environment, leading to the depicted rotamer being the most populated one. Source data are provided as a Source Data file.

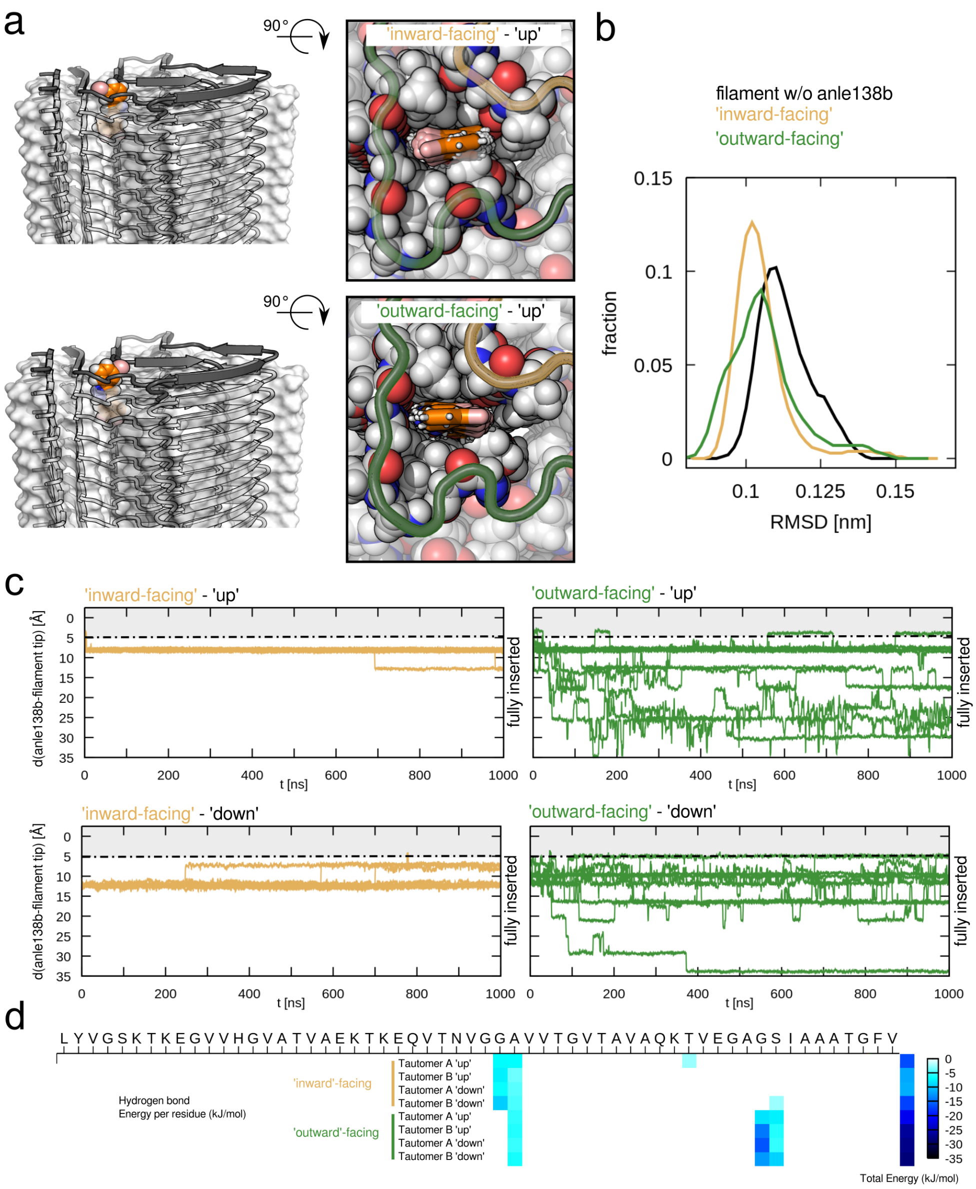

Supplementary Fig. 6. Translational motion and interactions of anle138b inside the α-synuclein L2 protofilament structure. a Top view of anle138b structure ensemble shown as overlay for representative trajectories of inward- and outward-facing binding poses**.** A colour code denotes the individual simulation sets for each binding pose in all following panels (inward-facing - yellow, outward-facing - green). **b** The α-synuclein cross-β protofilament structure was retained on the µs simulation time scale in the presence of the compound inside the cavity as shown by the comparison of the protofilament's main-chain and Cβ atom root mean square deviations (RMSD) for the simulation sets without (black) and with internally bound anle138b. **c** Stepwise translational motion of anle138b inside the cavity along the fibril axis i.e. the insertion depth of the compound with respect to the outermost β-strand layer is shown for all trajectories of the four principal binding poses. **d** Internal binding of anle138b inside the cavity of the α-synuclein protofilament structure results in polar interactions. The average hydrogen bond energies between anle138b and the backbone atoms of the α-synuclein protofilament structure are shown as a function of α-synuclein residue index. In terms of hydrogen bond energetics, the outward-facing anle138b poses show the most favorable hydrogen bonds to the β-sheet segment with residues G86 and S87 in either orientation (pointing 'up' or 'down' the protofilament axis) and irrespective of tautomeric form. Source data are provided as a Source Data file.

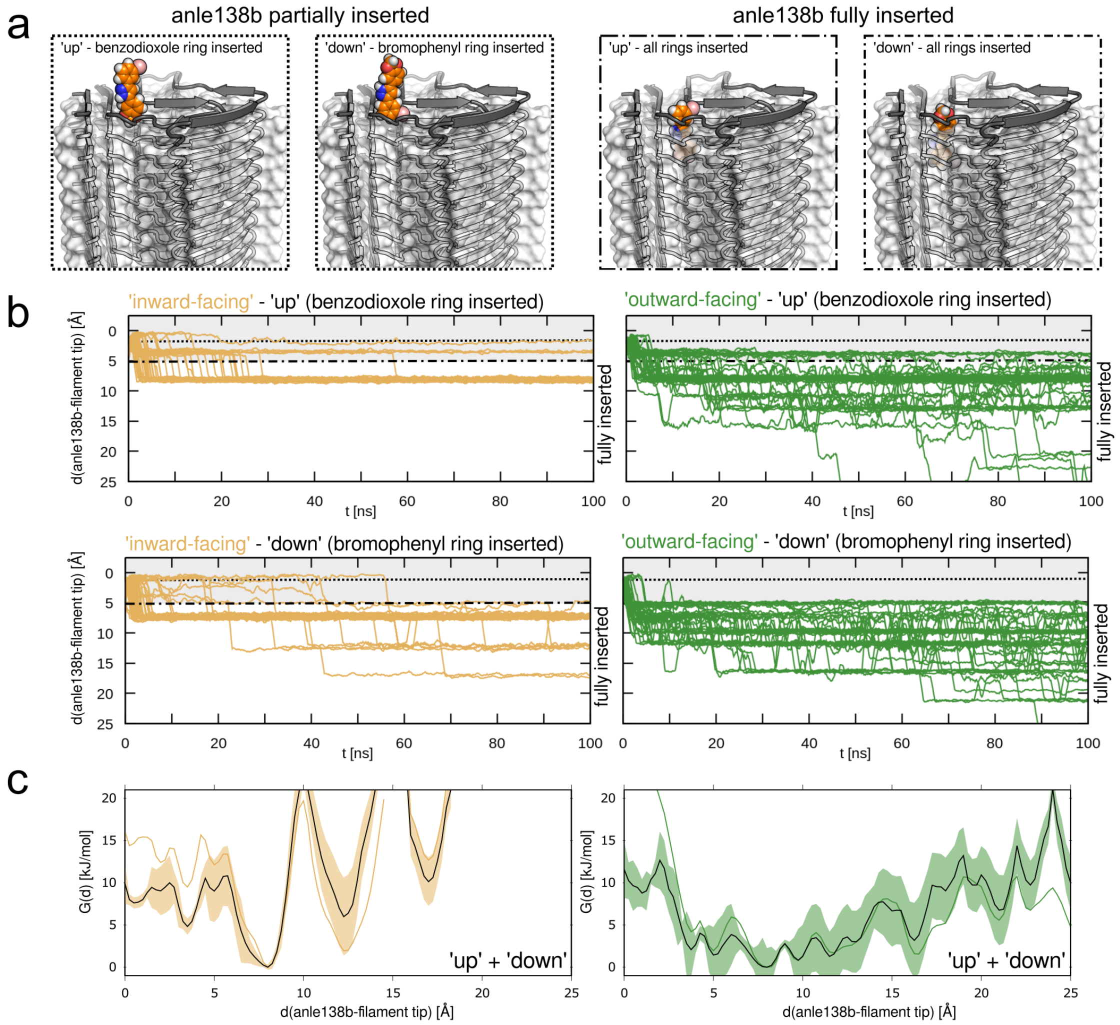

Supplementary Fig. 7. Testing of additional anle138b binding poses inside the α-synuclein protofilament. a Cryo-EM structure of α-synuclein protofilament L2 with anle138b modelled inside the cavity in four additional binding poses used as starting structures for the MD simulations: Here the compound was placed at the edge of the cavity in inward- or outward-facing binding poses, but with only one moiety flanking the pyrazole ring (either the bromophenyl or benzodioxole ring) buried inside the cavity. A colour code denotes the individual simulation sets for each binding pose in all following panels (inward-facing - yellow, outward-facing - green). Representative structures illustrate the starting poses and fully inserted anle138b molecules for the outward-facing binding poses. The latter are corresponding to an anle138b insertion depth of higher than 0.5 nm. Protein side chain atoms, as well as water and lipid molecules omitted for clarity in all renderings. **b** Anle138b readily migrated towards the center of the β-strand stack within 100 ns in 96 out of 100 trajectories, reaffirming that adopting the less solvent exposed, internal binding site is more favorable than the partially inserted conformation. **c** Binding profiles as function of anle138b insertion depth (black lines) are shown for inward-facing and outward-facing pose with error bars depicted by shading. Binding profiles for partially inserted starting poses (black lines) converge towards the ones obtained from the fully inserted (colored lines, see Fig. 3) starting poses. Data are presented as mean values ± SEM (indicated by shading). Source data are provided as a Source Data file.

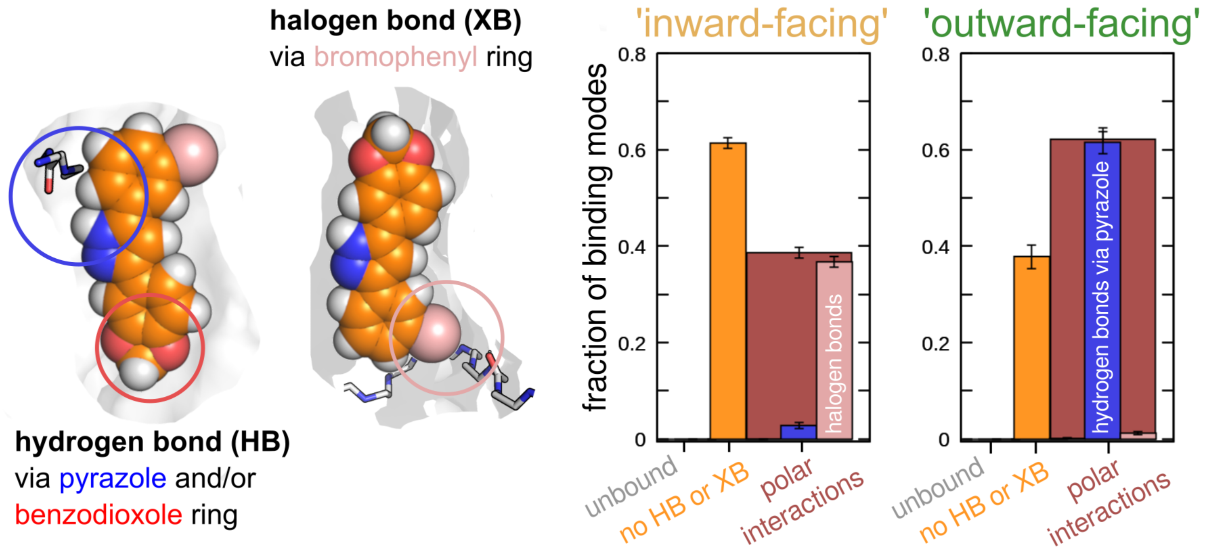

Supplementary Fig. 8. Binding modes observed in the MD simulations for internal anle138b poses. Interactions of internally bound anle138b with the α-synuclein protofilament were quantified as binding modes for inward- and outward-facing poses, respectively. Binding mode classification was based on polar interaction patterns i.e. through directional hydrogen bond formation via the pyrazole (blue) and/or benzodioxole (red) ring, as well as halogen bond interactions involving the bromine atom (pink), as observed in the MD simulations (see Methods). Data are presented as mean values ± SEM (indicated by error bars) and was derived from 20 independent simulations, respectively. Source data are provided as a Source Data file.

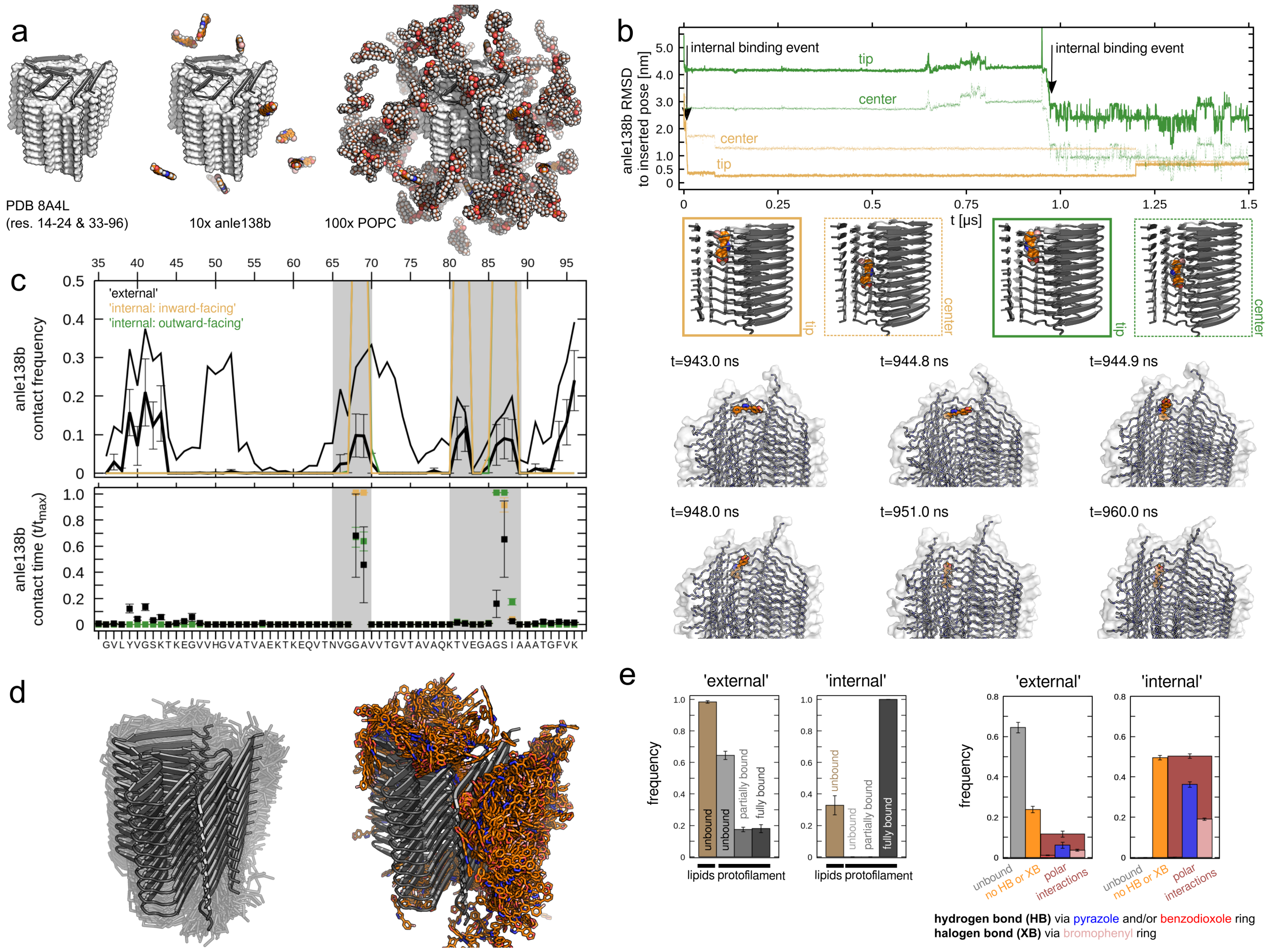

Supplementary Fig. 9. Analysis of anle138b binding to α-synuclein protofilament from random, non-inserted starting poses. a In an effort to further examine the high affinity of anle138b for the internal binding site and the influence of lipids on anle138b binding, we ran a total of 20 independent, 1.5 µs long simulations with anle138b starting randomly and distant from the α-synuclein protofilament model. The initial α-synuclein L2 protofilament model was simulated as stack of ten cross β-strand layers based on PDB ID 8A4L in the presence of 100 dispersed POPC molecules and with ten initially unbound anle138b molecules. A colour code denotes the individual simulation sets for each binding pose in the following panels (inward-facing - yellow, outward-facing - green). **b** Ligand RMSD for two, independent insertion events into the protofilament cavity observed from the same set of simulations. Both, inward- and outward-facing, internal binding poses were spontaneously visited at least once and with a ligand heavy atom RMSD of less than 0.2 nm with respect to both initially chosen, internal anle138b conformations. Representative structures are shown for one of the two independent trajectories. For clarity, ions as well as water and lipid molecules were omitted in the shown simulation snapshots. **c** Averaged anle138b contact frequencies to α-synuclein residues of the protofilament structure (thin lines: all β-strand layers considered; thick lines: excluding edge β-strand layers of the α-synuclein protofilament structure) are shown together with the average time of polar anle138b contacts to the protofilament backbone (normalized against trajectory length). Data are presented as mean values ± SEM (indicated by error bars) and derived from 20 independent simulations, respectively. A substantial amount of unspecific binding to the N- & C-terminal regions of the α-synuclein sequence, as well as the exposed surface of both protofilament tips was observed in the set of simulations with random, non-inserted starting poses. However, the highest average contact time of anle138b was found for residues G68, A69, G86 and S87 constituting the internal fibril cavity, while other residue contacts were not maintained as long. **d** Overlay of protofilament structure ensemble with bound anle138b spontaneously sampled during 10 independent MD

runs of 1.5 μs per system additionally shown. **e** Population of anle138b binding modes highlight the stark difference between the anle138b-protofilament interactions in the externally vs the internally bound state. The external anle138b molecules are predominantly lipid-bound (brown bar) and showed a high fraction of unbound (light grey bar) or only partially anle138b (grey bar) binding modes, as well as a decreased ratio of polar contacts involving hydrogen or halogen bonding to the protofilament (red brown bar), compared to the internally bound anle138b molecules. Data are presented as mean values ± SEM (indicated by error bars) and derived from 20 independent simulations, respectively. Source data are provided as a Source Data file.

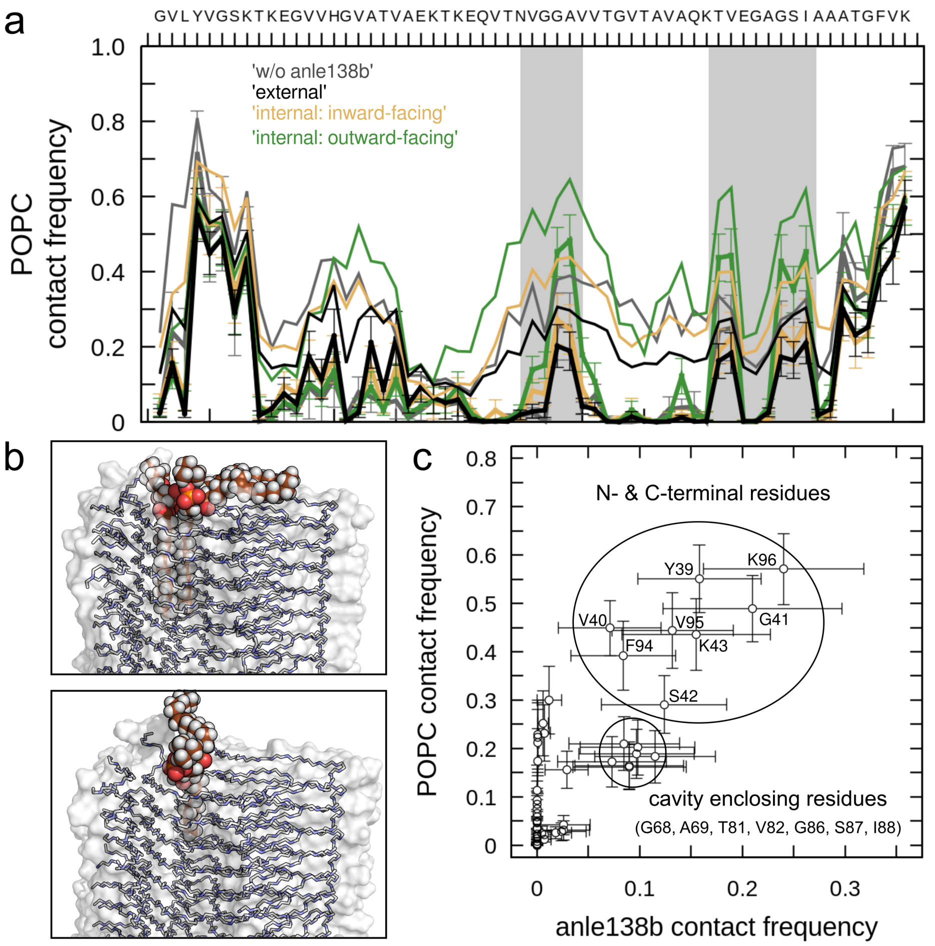

Supplementary Fig. 10. Analysis of lipid binding to α-synuclein protofilament from random, non-inserted starting poses. a Averaged POPC contact frequencies to α-synuclein residues of protofilament structure for simulation sets with and without anle138b (thin lines: all β-strand layers considered; thick lines: excluding edge β-strand layers of the α-synuclein protofilament structure). A colour code denotes the individual simulation sets for each binding pose in the following panels (inward-facing - yellow, outward-facing - green). Data are presented as mean values ± SEM (indicated by error bars) and has been derived from 20 independent simulations, respectively. Lipid binding to residues 38-42 as well as residues 93-95 of the α-synuclein fibril protofilament was recently shown by us[1, 2] and is also reproduced by the MD simulations in this study. In addition, our simulations show the frequent binding of individual aliphatic tails into the cavity, thus underscoring the highly hydrophobic nature of this part of the α-synuclein protofilament structure. **b** Representative structures for spontaneous insertion events of aliphatic lipid chains into the protofilament cavity from two independent simulations. On top, tails of two individual POPC molecules are shown inserted together, below one inserted tail of one POPC molecule is shown. For clarity, protein side chain atoms, as well as water and all non-inserted lipid molecules were omitted. **c** Correlation between averaged residue contact frequencies of anle138b and POPC molecules to the α-synuclein protofilament core (excluding edge layers). Data are presented as mean values ± SEM (indicated by error bars) derived from 40 independent simulations. A high correlation for POPC and anle138b contacts on the protofilament surface exists, except for the residues that constitute the internal binding site. Due to steric restrictions POPC lipid molecules cannot access the full length of the tubular cavity inside the α-synuclein protofilament and therefore are not able to bind the continuous internal binding site to the same extent as anle138b. Source data are provided as a Source Data file.

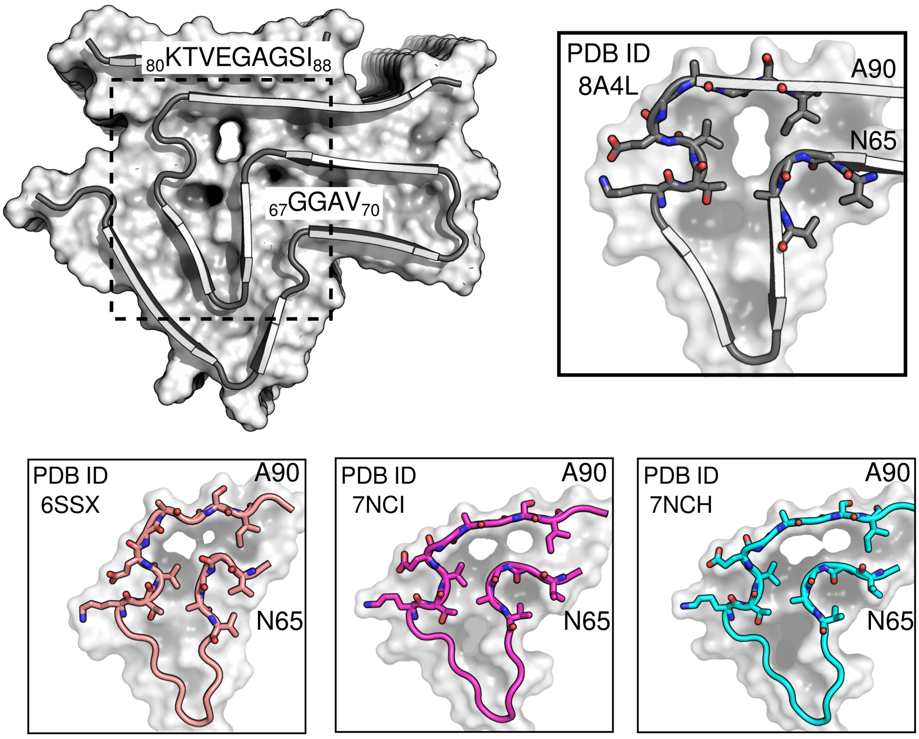

Supplementary Fig. 11. Tubular cavity conformation in polymorphic α-synuclein protofilament folds. The 3D structure of α-synuclein protofilament L2 (PDB ID 8A4L) grown in the presence of lipids as determined by cryo-EM[1]. Surface representation of residue stretch 65 to 90 highlights the location of the tubular cavity within the protofilament. The protofilament fold of the backbone is depicted in cartoon representation and the cavity enclosing residues 67-70 and 80-88 are shown with sticks. The same set of residues are shown for atomic structures of polymorph 2 α-synuclein protofilaments grown in the absence of lipids: 6SSX/6RT0[3], 7NCI and 7NCH[4].

Supplementary Tables

Supplementary Table 1: Details of NHHC experiments for the identification of contacts.

Sample	MAS (kHz)	Sample Temp. (K)	Enhancement factor	Meas. Time (h)	No. scans*No. experiments	Recycle delay (s)
u-^{13}C,^{15}N	12.5	114	6-9	91.5	1280*4	2.0
^{13}C,^{15}N-Ile	9.5	104	7-8	77.3	256*13	2.5
^{13}C,^{15}N-Leu, Tyr	12.5	114	7	192.5	2048*8	1.75
^{13}C-Phe	12.5	114	7	68.7	1024*3	2.5
^{13}C, ^{15}N-Gly	12.5	114	13	120.0	640*5	4.2

Supplementary Table 2: Experimental parameters for the FS-REDOR experiment.

	90°-pulse (Power / Width)	CP (Power/ Width)	Hard 180°-pulse (Power / Width)	Selective (Q3) 180°-pulse[1] (Width / Carrier Frequency position)
^{1}H	100 kHz / 2.5 μs	[95-106] (kHz) / 1.5 ms	-	-
^{13}C	-	71 kHz / 1.5 ms	-	2068.97 μs / 26 ppm
^{15}N	-	-	41.67 kHz / 12 μs	965.52 μs / 195 ppm

[1] Selective Q3.1000 shape pulses[5] were used from the standard Bruker library.

Supplementary Table 3: Summary of performed simulations. For each studied system the number of independent MD simulation replicates and length of the trajectories per simulation condition is listed.

Simulation system	Initial binding pose/starting position of anle138b molecules	No. of replicates and length of trajectories
10 β-strand layers, 100 POPC lipids, 41k waters	without anle138b	10 × 1.0 μs
10 β-strand layers, 100 POPC lipids, 41k waters	1 × Tautomer A, fully bound inside the protofilament cavity, bromophenyl-ring pointing 'up', pyrazole nitrogens facing 'inward'	5 × 1.0 μs
10 β-strand layers, 100 POPC lipids, 41k waters	1 × Tautomer B, fully bound inside the protofilament cavity, bromophenyl-ring pointing 'up', pyrazole nitrogens facing 'inward'	5 × 1.0 μs
10 β-strand layers, 100 POPC lipids, 41k waters	1 × Tautomer A, fully bound inside the protofilament cavity, bromophenyl-ring pointing 'down', pyrazole nitrogens facing 'inward'	5 × 1.0 μs
10 β-strand layers, 100 POPC lipids, 41k waters	1 × Tautomer B, fully bound inside the protofilament cavity, bromophenyl-ring pointing 'down', pyrazole nitrogens facing 'inward'	5 × 1.0 μs
10 β-strand layers, 100 POPC lipids, 41k waters	1 × Tautomer A, fully bound inside the protofilament cavity, bromophenyl-ring pointing 'up', pyrazole nitrogens facing 'outward'	5 × 1.0 μs
10 β-strand layers, 100 POPC lipids, 41k waters	1 × Tautomer B, fully bound inside the protofilament cavity, bromophenyl-ring pointing 'up', pyrazole nitrogens facing 'outward'	5 × 1.0 μs
10 β-strand layers, 100 POPC lipids, 41k waters	1 × Tautomer A, fully bound inside the protofilament cavity, bromophenyl-ring pointing 'down', pyrazole nitrogens facing 'outward'	5 × 1.0 μs
10 β-strand layers, 100 POPC lipids, 41k waters	1 × Tautomer B, fully bound inside the protofilament cavity, bromophenyl-ring pointing 'down', pyrazole nitrogens facing 'outward'	5 × 1.0 μs
10 β-strand layers, 100 POPC lipids, 41k waters	1 × Tautomer A, partially bound at the edge of the protofilament cavity, bromophenyl-ring pointing 'up', pyrazole nitrogens facing 'inward'	25 × 100 ns
10 β-strand layers, 100 POPC lipids, 41k waters	1 × Tautomer B, partially bound at the edge of the protofilament cavity, bromophenyl-ring pointing 'up', pyrazole nitrogens facing 'inward'	25 × 100 ns
10 β-strand layers, 100 POPC lipids, 41k waters	1 × Tautomer A, partially bound at the edge of the protofilament cavity, bromophenyl-ring pointing 'down', pyrazole nitrogens facing 'inward'	25 × 100 ns
10 β-strand layers, 100 POPC lipids, 41k waters	1 × Tautomer B, partially bound at the edge of the protofilament cavity, bromophenyl-ring pointing 'down', pyrazole nitrogens facing 'inward'	25 × 100 ns
10 β-strand layers, 100 POPC lipids, 41k waters	1 × Tautomer A, partially bound at the edge of the protofilament cavity, bromophenyl-ring pointing 'up', pyrazole nitrogens facing 'outward'	25 × 100 ns
10 β-strand layers, 100 POPC lipids, 41k waters	1 × Tautomer B, partially bound at the edge of the protofilament cavity, bromophenyl-ring pointing 'up', pyrazole nitrogens facing 'outward'	25 × 100 ns
10 β-strand layers, 100 POPC lipids, 41k waters	1 × Tautomer A, partially bound at the edge of the protofilament cavity, bromophenyl-ring pointing 'down', pyrazole nitrogens facing 'outward'	25 × 100 ns
10 β-strand layers, 100 POPC lipids, 41k waters	1 × Tautomer B, partially bound at the edge of the protofilament cavity, bromophenyl-ring pointing 'down', pyrazole nitrogens facing 'outward'	25 × 100 ns
10 β-strand layers, 100 POPC lipids, 41k waters	10 × Tautomer A, randomly placed in solvent	10 × 1.5 μs
10 β-strand layers, 100 POPC lipids, 41k waters	10 × Tautomer B, randomly placed in solvent	10 × 1.5 μs

References

1. Frieg B, et al. The 3D structure of lipidic fibrils of α-synuclein. *bioRxiv*, 2022.2003.2002.481946 (2022).
2. Antonschmidt L, et al. Insights into the molecular mechanism of amyloid filament formation: Segmental folding of α-synuclein on lipid membranes. *Sci Adv* **7**, eabg2174 (2021).
3. Guerrero-Ferreira R, et al. Two new polymorphic structures of human full-length alpha-synuclein fibrils solved by cryo-electron microscopy. *eLife* **8**, e48907 (2019).
4. Lövestam S, et al. Seeded assembly in vitro does not replicate the structures of α-synuclein filaments from multiple system atrophy. *FEBS Open Bio* **11**, 999-1013 (2021).
5. Emsley L, Bodenhausen G. Optimization of shaped selective pulses for NMR using a quaternion description of their overall propagators. *J. Magn. Reson.* (1969) **97**, 135-148 (1992).
