# Supplementary material for: The clinical drug candidate anle138b binds in a cavity of lipidic α-synuclein fibrils
Source: Nat Commun. 2022 Sep 14;13:5385. doi: 10.1038/s41467-022-32797-w (PMC9474542; doi:10.1038/s41467-022-32797-w)
Supplement: Supplementary file 2 — Description of additional supplementary files [file 41467_2022_32797_MOESM2_ESM.pdf]

Description of Additional Supplementary Files

Supplementary Movie S1 : **Translational motion of anle138b for the outward-facing, internal binding pose.** The movie shows the first 850 ns of a representative MD trajectory for one anle138b molecule modelled inside the internal cavity of an αsynuclein protofilament L2 structure, consisting of ten β-strand layers. The protofilament is shown simultaneously from the side (left) and down the filament axis (right). The protofilament structure is initially shown in surface representation. For clarity, parts of the structure were hidden in successive manner such that only the protein backbone and Cβ-atoms remain visible. The two residue stretches enclosing the internal cavity (67GGAV70 and 80KTVEGAGSI88) are initially highlighted by spheres and later by white colored sticks. At all times, the anle138b molecule is shown with spheres and orange colored carbon atoms, water and lipid molecules were omitted.
