# Supplementary material for: The clinical drug candidate anle138b binds in a cavity of lipidic α-synuclein fibrils
Source: Nat Commun. 2022 Sep 14;13:5385. doi: 10.1038/s41467-022-32797-w (PMC9474542; doi:10.1038/s41467-022-32797-w)
Supplement: Supplementary file 4 — Reporting Summary [file 41467_2022_32797_MOESM4_ESM.pdf]

nature research

Corresponding author(s): Bert L. de Groot, Christian Griesinger, Loren B. Andreas

Last updated by author(s): 2022/07/25

Reporting Summary

Nature Research wishes to improve the reproducibility of the work that we publish. This form provides structure for consistency and transparency in reporting. For further information on Nature Research policies, see our Editorial Policies and the Editorial Policy Checklist.

Statistics

For all statistical analyses, confirm that the following items are present in the figure legend, table legend, main text, or Methods section.

n/a	Confirmed	
☐	☒	The exact sample size (*n*) for each experimental group/condition, given as a discrete number and unit of measurement
☐	☒	A statement on whether measurements were taken from distinct samples or whether the same sample was measured repeatedly
☒	☐	The statistical test(s) used AND whether they are one- or two-sided<br>*Only common tests should be described solely by name; describe more complex techniques in the Methods section.*
☒	☐	A description of all covariates tested
☒	☐	A description of any assumptions or corrections, such as tests of normality and adjustment for multiple comparisons
☐	☒	A full description of the statistical parameters including central tendency (e.g. means) or other basic estimates (e.g. regression coefficient) AND variation (e.g. standard deviation) or associated estimates of uncertainty (e.g. confidence intervals)
☒	☐	For null hypothesis testing, the test statistic (e.g. *F*, *t*, *r*) with confidence intervals, effect sizes, degrees of freedom and *P* value noted<br>*Give P values as exact values whenever suitable.*
☒	☐	For Bayesian analysis, information on the choice of priors and Markov chain Monte Carlo settings
☒	☐	For hierarchical and complex designs, identification of the appropriate level for tests and full reporting of outcomes
☒	☐	Estimates of effect sizes (e.g. Cohen's *d*, Pearson's *r*), indicating how they were calculated

Our web collection on statistics for biologists contains articles on many of the points above.

Software and code

Policy information about availability of computer code

Data collection	NMR experiments: Bruker Topspin (different versions);<br>MD simulations: GROMACS 2018.3(including implementations of P-LINCS, SETTLE, non-bonded Verlet scheme, PME, velocity-rescale Temperature coupling and Parrinello-Rahman barostat); AMBER99SB*-ILDN; Ion parameters (Dang et al.); TIP3P water model; modified version of the all-atom Slipid force field (Melcr et al.); GAFF parameters (anle138b, Matthes et al.)
Data analysis	NMR experiments: CcpNmr; NMRFAM-Sparky; NMRPipe; Origin(Pro) 8.5;<br>MD simulations: Custom Python(v 3.9.7), Awk and Bash scripts were used to postprocess output from GROMACS 2018.3 analysis tools (gmx hbond & gmx mindist) and g_contacts (Blau et al.) used to calculate interatomic distances; Fortran code was used to obtain hydrogen bond energies (Espinosa et al.)

For manuscripts utilizing custom algorithms or software that are central to the research but not yet described in published literature, software must be made available to editors and reviewers. We strongly encourage code deposition in a community repository (e.g. GitHub). See the Nature Research guidelines for submitting code & software for further information.

Data

Policy information about availability of data

All manuscripts must include a data availability statement. This statement should provide the following information, where applicable:

- Accession codes, unique identifiers, or web links for publicly available datasets
- A list of figures that have associated raw data
- A description of any restrictions on data availability

NMR spectra raw data generated in this study and mass spectrometry data for the synthesis of selectively labelled α-synuclein have been deposited in the open research data repository Edmond at https://doi.org/10.17617/3.9C6TEW. The molecular dynamics data generated in this study have been deposited in the Edmond

April 2020

data repository at https://doi.org/10.17617/3.R4FJQG. Assigned chemical shift data (HN, Cα, Cβ, and C') for α-synuclein fibrils were deposited in the BMRB as updates under the accession number 50585 (https://doi.org/10.13018/BMR50585). Atomic coordinates for L2A-fibrils of αSyn used for MD-simulations and visualization in this manuscript are available in the Protein Data Bank under the accession code 8A4L (https://www.rcsb.org/structure/unreleased/8A4L). Previously published structures of αSyn fibrils used for a comparison of tubular fibril cavities are available in the Protein Data Bank under the accession codes 6SSX (http://doi.org/10.2210/pdb6SSX/pdb) , 7NCI (http://doi.org/10.2210/pdb7NCI/pdb) and 7NCH (http://doi.org/10.2210/pdb7NCH/pdb). All other data generated or analysed during this study are included in this published article (and its supplementary information files). Source data are provided with this paper.

Field-specific reporting

Please select the one below that is the best fit for your research. If you are not sure, read the appropriate sections before making your selection.

☒ Life sciences ☐ Behavioural & social sciences ☐ Ecological, evolutionary & environmental sciences

For a reference copy of the document with all sections, see nature.com/documents/nr-reporting-summary-flat.pdf

Life sciences study design

All studies must disclose on these points even when the disclosure is negative.

Sample size	NMR experiments: (number of repetitions, number of time points in the indirect dimensions) were adapted to the signal to noise obtained. Differerences originate from varying fibril sample yield and losses in the rotor filling process. Measurements were continued until sufficient signal to noise was obtained. MD simulations: (number of simulation replicates and simulation length) were chosen such that are results for each simulation condition are statistical significant according to the standard error of the mean
Data exclusions	no data were excluded
Replication	MD simulation: For each simulation condition we simulated between 10 to 25 replicates. All anle138b poses starting from either fully or partially bound conditions did not unbind from the internal protofilament cavity (all replication attempts successful - 140 of 140). Spontaneous binding from anle138b to the internal cavity from initially random positions in the solvent was observed in 2 of 20 simulations (at least one replication attempt successful).
Randomization	no randomization was applied in NMR samples as demands on protein material are already high. MD simulations were tested with randomized anle138b starting poses.
Blinding	no blinding. Adjustment of NMR parameters requires prior knowledge of isotope labeling. The anyway long measurement times (days to weeks) would be multiplied if parameters are incorrect.

Reporting for specific materials, systems and methods

We require information from authors about some types of materials, experimental systems and methods used in many studies. Here, indicate whether each material, system or method listed is relevant to your study. If you are not sure if a list item applies to your research, read the appropriate section before selecting a response.

Materials & experimental systems

n/a	Involved in the study	
☒	☐	Antibodies
☒	☐	Eukaryotic cell lines
☒	☐	Palaeontology and archaeology
☒	☐	Animals and other organisms
☒	☐	Human research participants
☒	☐	Clinical data
☒	☐	Dual use research of concern

Methods

n/a	Involved in the study	
☒	☐	ChIP-seq
☒	☐	Flow cytometry
☒	☐	MRI-based neuroimaging
